# In vivo nuclear RNA structurome reveals RNA-structure regulation of mRNA processing in plants

**DOI:** 10.1186/s13059-020-02236-4

**Published:** 2021-01-04

**Authors:** Zhenshan Liu, Qi Liu, Xiaofei Yang, Yueying Zhang, Matthew Norris, Xiaoxi Chen, Jitender Cheema, Huakun Zhang, Yiliang Ding

**Affiliations:** 1 Department of Cell and Developmental Biology, John Innes Centre, Norwich Research Park, Norwich, NR4 7UH UK; 2 Key Laboratory of Molecular Epigenetics of the Ministry of Education, Northeast Normal University, Changchun, 130024 China

**Keywords:** mRNA processing, Splicing, Polyadenylation, RNA structure, SHAPE

## Abstract

**Background:**

mRNA processing is critical for gene expression. A challenge in regulating mRNA processing is how to recognize the actual mRNA processing sites, such as splice and polyadenylation sites, when the sequence content is insufficient for this purpose. Previous studies suggested that RNA structure affects mRNA processing. However, the regulatory role of RNA structure in mRNA processing remains unclear.

**Results:**

Here, we perform in vivo selective 2′-hydroxyl acylation analyzed by primer extension (SHAPE) chemical profiling on *Arabidopsis* and generate the in vivo nuclear RNA structure landscape. We find that nuclear mRNAs fold differently from cytosolic mRNAs across translation start and stop sites. Notably, we discover a two-nucleotide single-stranded RNA structure feature upstream of 5′ splice sites that is strongly associated with splicing and the selection of alternative 5′ splice sites. The regulatory role of this RNA structure feature is further confirmed by experimental validation. Moreover, we find the single-strandedness of branch sites is also associated with 3′ splice site recognition. We also identify an RNA structure feature comprising two close-by single-stranded regions that is specifically associated with both polyadenylation and alternative polyadenylation events.

**Conclusions:**

We successfully identify pre-mRNA structure features associated with splicing and polyadenylation at whole-genome scale and validate an RNA structure feature which can regulate splicing. Our study unveils a new RNA structure regulatory mechanism for mRNA processing.

## Background

In eukaryotes, mRNAs undergo several processing steps including 5′ capping, splicing, and 3′ cleavage/polyadenylation to become functional mature mRNAs. Thus, mRNA processing plays a critical role during gene expression [1, 2]. Over past decades, a key question is how mRNA processing sites, such as polyadenylation and splice sites, are precisely recognized in the transcriptome, particularly from surrounding sites with similar sequence content [3, 4]. For instance, 5′ splice site recognition was found to be not always dependent on the sequence content of U1 snRNA binding motif. Some 5′ splice sites were selected over those flanking sites with better complementarity to U1 snRNA binding sequence [4]. In case-by-case studies, quite a number of RNA binding proteins have been identified that contribute to the recognition of actual polyadenylation and splice sites [4, 5]. However, a general regulatory mechanism that recognizes actual sites during mRNA processing is lacking. As an intrinsic characteristic of RNA molecules, RNA structure was suggested to be involved in mRNA processing [6]. Previous individual studies suggested that RNA structure can affect polyadenylation and splicing [7–13]. Yet, how RNA structure contributes to the recognition of polyadenylation and splice sites, in general, remains elusive.

With recent advances in RNA structure profiling [14–16], more attention has been drawn toward understanding how RNA structure influences mRNA processing. Previous in vitro enzymatic RNA structure profiling (utilizing RNases that selectively cleave either single-stranded or double-stranded nucleotides) in *Arabidopsis* nuclear RNAs found that the 5′ end of introns was more double-stranded compared to upstream exons, and the 3′ end of introns was more single-stranded compared to upstream intron regions [14]. However, no significant structure signatures were identified for either polyadenylation or alternative polyadenylation sites [14]. This may be due to limitations imposed by using RNases, which are quite bulky and less sensitive in detecting specific RNA structures, compared to the relatively small chemicals used for RNA structure probing [17, 18]. Furthermore, several previous studies have shown that in vitro RNA structures were not able to reflect the proper folding status of RNAs in living cells [19, 20]. A recent in vivo dimethyl sulfate (DMS) RNA structure profiling study on human mature mRNAs identified RNA structure features for polyadenylation (poly(A)) sites [15]. A more folded structure downstream of the polyadenylation signal motif was identified that facilitated polyadenylation [15]. However, mammalian RNAs were found to adopt different structure conformations in different cellular compartments [21]. Thus, the structure of mature mRNAs in the cytosol is likely to be different from the structure of pre-mRNA in the nucleus. If so, mature mRNA structures are unlikely to reveal the role of RNA structure in polyadenylation. A notable limitation of this DMS method is the loss of RNA structure information for the half transcriptome because DMS only detects structure information of As (Adenines) and Cs (Cytosines), lacking the base-pairing status of Us (Uracils) and Gs (Guanines).

Here, we studied the role of RNA structure in mRNA processing by performing in vivo SHAPE (*S*elective 2′ *H*ydroxyl *A*cylation analyzed by *P*rimer *E*xtension) chemical probing on *Arabidopsis thaliana* nuclear RNAs, to generate the first in vivo RNA structure landscape with all four nucleotides in plants. We found that nuclear mRNA structures are globally different from cytosolic mRNA structures in *Arabidopsis*. Our study further successfully dissected pre-mRNA structure features before mRNA processing and determined the regulatory role of RNA structure during mRNA maturation.

## Results

### Nuc-SHAPE-Structure-Seq generates in vivo RNA structure landscape of *Arabidopsis* nuclear RNAs with high coverage and accuracy

**Fig. 1:**
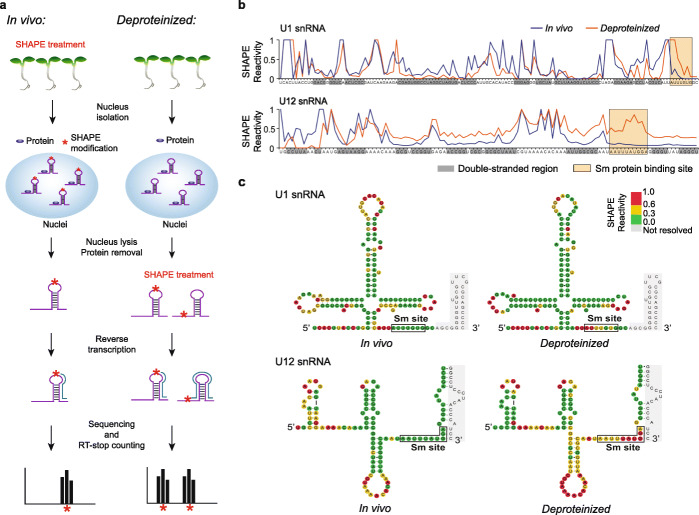
Nuc-SHAPE-Structure-Seq method can accurately probe the in vivo RNA structure of nuclear RNAs. **a** Schematic pipeline of Nuc-SHAPE-Structure-Seq for both in vivo and *deproteinized* conditions. Asterisks, SHAPE modification; blue oval, protein; RT, reverse transcription. For in vivo treatments (left), NAI was applied to *Arabidopsis thaliana* seedlings directly and single-stranded nucleotides of RNA were modified. SHAPE treatment was also applied on the RNAs after nuclei isolation and protein removal, which we termed the “*deproteinized* condition” (right). Deep sequencing was performed followed by the RT-stop counting. **b** SHAPE reactivity profiles of U1 and U12 snRNAs. SHAPE reactivity profiles of both in vivo (blue) and *deproteinized* (orange) conditions were shown. Double-stranded regions were shaded with gray. Sm protein binding sites were highlighted with yellow boxes. At Sm protein binding sites, significantly higher SHAPE reactivities were observed under the *deproteinized* condition rather than the in vivo condition for both U1 and U12 snRNAs (paired *t* test, *P* value = 6.8e−3 and 3.1e−6 for U1 and U12 snRNA, respectively). **c** SHAPE reactivities are consistent with the phylogenetically derived U1 and U12 snRNA structures. Sm protein binding sites were highlighted with black boxes. Nucleotides were color-coded according to in vivo and *deproteinized* SHAPE reactivity values (SHAPE reactivity 0.6–1.0 marked in red, 0.3–0.6 marked in yellow, 0–0.3 marked in green)

To investigate the role of RNA structure in mRNA processing, we performed SHAPE chemical probing [22] on *Arabidopsis* and generated the first in vivo RNA structure profiles with all four nucleotides in plants. Firstly, SHAPE reagent (2-methylnicotinic acid imidazolide, NAI) treatment was applied on 5-day-old *Arabidopsis* seedlings [22] (Fig. 1a). Intact nuclei were isolated and nuclear RNAs were extracted. The intactness of isolated nuclei was confirmed by microscopy imaging with DAPI staining [23] (Additional file 1: Figure S1a). Enrichment of nuclear histone H3 protein and absence of cytoplasmic protein PEPC (phosphoenolpyruvate carboxylase) in the isolated nucleus further confirmed the high purity and quality of the isolated nuclei (Additional file 1: Figure S1b). We generated two independent biological replicates of (+)SHAPE (samples with SHAPE treatment) and (−)SHAPE (control samples without SHAPE treatment) Structure-Seq libraries for high-throughput sequencing [24, 25], and named our method Nuc-SHAPE-Structure-Seq (Fig. 1a, Additional file 1: Figure S2, see the “Methods” section). Given that interactions between RNA and RNA binding proteins can prevent the SHAPE modification, we also performed SHAPE treatment on nuclear RNAs after removing proteins thus generating *deproteinized* Nuc-SHAPE-Structure-Seq libraries in parallel (see the “Methods” section, Fig. 1a) to assess any effect on SHAPE modification signals caused by protein protection. Over 616 million 100-bp paired-end reads per library were generated and further mapped onto *Arabidopsis* genome sequences (TAIR10) with additional alternative spliced isoforms annotated from AtRTD2 database [26] (Additional file 1: Table S1).

Nucleotide modification in both (+)SHAPE and (−)SHAPE libraries was highly concordant, with slight enrichment in (+)SHAPE shown for As and Us over Cs and Gs, as expected, since As and Us tend to be more single-stranded than Cs and Gs (Additional file 1: Figure S3a). The high correlation of mRNA abundance between the two biological replicates indicated the high reproducibility of our Nuc-SHAPE-Structure-Seq libraries (Additional file 1: Figure S3b). To further validate the reproducibility of our SHAPE structure probing, we compared SHAPE reactivity profiles of two small nuclear RNAs with known secondary structure, U1 and U12 snRNA, between the two biological replicates and noted a high correlation between them (Pearson’s correlation coefficient = 0.93–0.97) (Additional file 1: Figure S3c). Thus, we merged these two biological replicates for further RNA structure analysis.

We assessed both the sequencing read coverage and reverse-transcription stop counts of our Nuc-SHAPE-Structure-Seq libraries. Notably, more than 20,752 genes had at least 10 reads per nucleotide coverage (Additional file 1: Figure S4a), among which more than 12,366 genes reached the threshold of at least one reverse-transcription stop (RT-stop) count per nucleotide for RNA structure analysis (Additional file 1: Figure S4b). Furthermore, we calculated the Pearson correlation coefficient (PCC) of the SHAPE reactivities between replicates for each mRNA and plotted the corresponding average PCC as a function of the RT-stop read coverage (RT-stop counts per nucleotide) (Additional file 1: Figure S5). The average PCC values are 0.87 and 0.89 for mRNAs with more than one RT-stop count per nucleotide in in vivo nuclear and cytosolic SHAPE-Structure-Seq libraries, respectively (Additional file 1: Figure S5). The high correlations between independent biological replicates indicate the high reproducibility of our SHAPE-Structure-Seq libraries (Additional file 1: Figure S5). To assess the accuracy of our RNA structure profiling, we compared SHAPE reactivity profiles of U1 and U12 snRNAs with their phylogenetically derived structures, which are evolutionarily conserved structures and are the closest models of in vivo structure [22, 27]. Overall, the SHAPE reactivities were consistent with phylogenetically derived RNA structures where high SHAPE reactivities were observed in single-stranded regions, while low SHAPE reactivities were at double-stranded nucleotides (Fig. 1b, c, Additional file 1: Table S2). Both U1 and U12 snRNAs interact with Sm proteins to form small nuclear ribonucleoparticle structures [22, 27]. We also found that SHAPE reactivities at Sm protein binding sites of U1 and U12 snRNA were significantly higher in the *deproteinized* rather than in vivo condition (Fig. 1b, c), suggesting that absence of protein protection in the *deproteinized* condition allowed nucleotide modification by SHAPE. To further confirm that our Nuc-SHAPE-Structure-Seq and SHAPE-Structure-Seq libraries can provide accurate in vivo RNA structure information, we compared the SHAPE reactivity profiles with three previously reported RNA structure models: (1) *THIC* pre-mRNA structure (TPP riboswitch), (2) the RNA structure of 5′UTR of *PSBA* mRNA (*ATCG00020*), and (3) the RNA structure of 5′UTR of *GRP3S* mRNA (*AT2G05380*). We found that our SHAPE reactivity profiles agree well with these previously reported individual RNA structure models (Additional file 1: Figure S6) [22, 28]. Collectively, these results indicated that our Nuc-SHAPE-Structure-Seq method can accurately probe in vivo RNA structures of nuclear RNAs.

### Nuclear mRNAs showed different RNA structure features from cytosolic mRNAs across translation start and stop sites

**Fig. 2:**
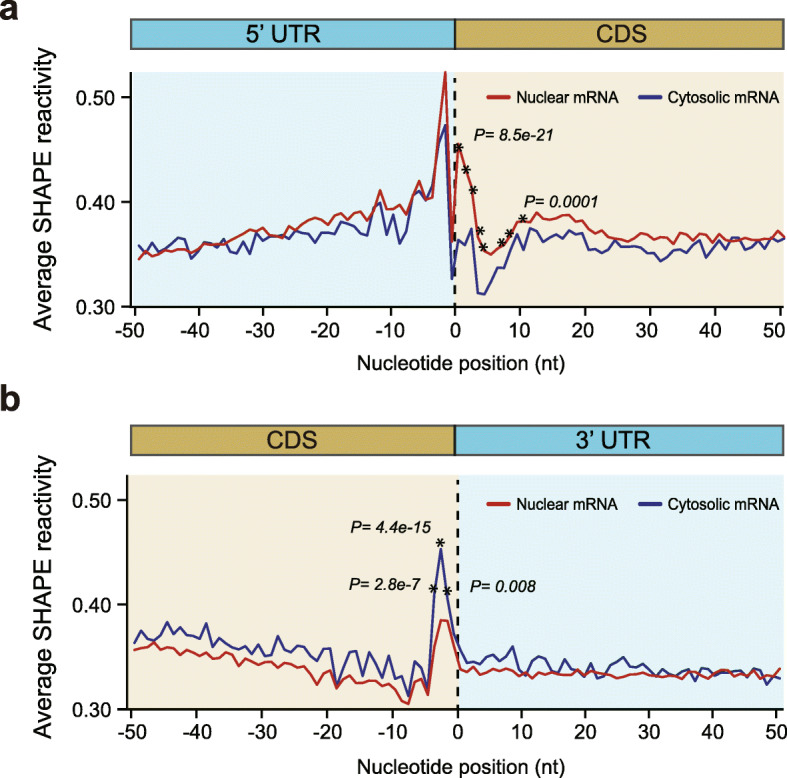
In vivo nuclear mRNA structures are different from cytosolic mRNA structures across translation start and stop sites. **a** Comparison of average SHAPE reactivity profiles between nuclear and cytosolic mRNAs across the translation start codon. Average SHAPE reactivities downstream of the start codon are significantly higher in nuclear mRNAs compared to cytosolic mRNAs (Mann-Whitney test, the highest and lowest *P* values for the first ten nucleotides of the CDS region are shown). **b** Comparison of SHAPE reactivity profiles between nuclear and cytosolic mRNAs across the translation stop codon. Average SHAPE reactivities at the stop codon are significantly lower in nuclear mRNAs compared to cytosolic mRNAs (Mann-Whitney test, the *P* values were shown)

Cytosolic mRNAs are the processed products from nuclear mRNAs; thus, they share the same sequences. However, whether they share the same RNA structure features remains unclear. To address this question, we generated in vivo SHAPE-Structure-Seq libraries of *Arabidopsis* cytosolic mRNAs in parallel. We then compared these libraries with our in vivo Nuc-SHAPE-Structure-Seq libraries. Previous studies on total mRNAs dominated by cytosolic mRNAs observed unique structure features across translation start and stop codons that were associated with translation [24, 29–31]. Thus, we examined the SHAPE reactivity patterns across these two sites. Consistent with the previous observations, we also found higher SHAPE reactivities upstream of start codons, lower SHAPE reactivities downstream of start codons, and higher SHAPE reactivities at stop codons compared to flanking regions in our cytosolic SHAPE-Structure-Seq libraries (Fig. 2a, b), which further confirmed the reliability of our SHAPE-Structure-Seq libraries. We then compared the SHAPE reactivities between nuclear and cytosolic mRNAs across these two sites. Significantly higher SHAPE reactivities downstream of start codons and significantly lower SHAPE reactivities at stop codons in nuclear mRNAs were observed compared to those in cytosolic mRNAs, whereas no such significant differences were observed at the flanking regions (Fig. 2a, b). Thus, our results suggest nuclear mRNAs fold differently from cytosolic mRNAs across translation start and stop sites, which implies nuclear and cytosolic mRNAs might adopt different structures to serve their respective biological functions, e.g., translation in the cytosol and mRNA processing in the nucleus. Therefore, we further investigated how nuclear mRNA structures are associated with mRNA processing.

### Distinctive pre-mRNA structure features are strongly associated with both splicing and alternative splicing

Splicing is a key mRNA processing step that was previously suggested to be influenced by RNA structure [8]. Since only pre-mRNA structure before splicing (unspliced primary transcripts) can be used for dissecting the mechanism underpinning splicing, we firstly assessed whether pre-mRNAs were enriched in our Nuc-SHAPE-Structure-Seq data. We found that the expression abundance of constitutively spliced introns was much higher in our Nuc-SHAPE-Structure-Seq libraries compared to cytosolic SHAPE-Structure-Seq libraries, indicating high enrichment of pre-mRNAs in Nuc-SHAPE-Structure-Seq data (Additional file 1: Figure S7). Since nuclear mRNAs still contain spliced transcripts, we only used reads mapped across exon-intron junctions and in intron regions in SHAPE reactivity calculation to obtain RNA structure information of pre-mRNAs before splicing (see details in the “Methods” section). Also, to eliminate any ambiguous read assignment at the conserved dinucleotide AG at 3′ splice sites (3′ss), we only calculated SHAPE reactivities across 5′ splice sites (5′ss) and the whole intron except for AG at 3′ss (see details in the “Methods” section).

**Fig. 3:**
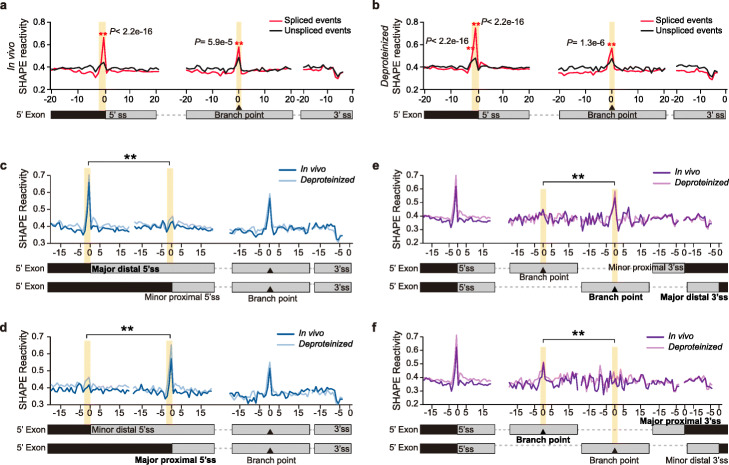
pre-mRNA secondary structure features upstream of 5′ss and at the branch site are associated with splicing and alternative splice site selection. **a**, **b** SHAPE reactivity profiles across 5′ss, branch point, and 3′ss for in vivo (**a**) and *deproteinized* (**b**) conditions. Average SHAPE reactivity profiles for spliced (red) versus unspliced (black) events are shown. Significantly higher SHAPE reactivities are observed at the − 1 and − 2 nt positions of 5′ss and the branch site for spliced events rather than unspliced events (marked with asterisks, Mann-Whitney test, *P* values are shown). **c** SHAPE reactivity profiles for alternative 5′ss events with the distal 5′ss as the major one. Average SHAPE reactivity profiles of both in vivo (dark blue) and *deproteinized* (light blue) conditions are shown. Significantly higher SHAPE reactivities only appear at − 1 and − 2 positions upstream of the major distal 5′ss rather than the minor proximal 5′ss (Mann-Whitney test, *P* value = 1.6e−4 and < 2.2e−16 at − 1 and − 2 positions under in vivo condition; *P* value = 6.1e−9 and < 2.2e−16 at − 1 and − 2 positions under *deproteinized* condition). **d** SHAPE reactivity profiles for alternative 5′ss events with the proximal 5′ss as the major one. The significantly higher SHAPE reactivities of − 1 and − 2 positions only appear upstream of the major proximal 5′ss rather than the minor distal 5′ss (Mann-Whitney test, *P* value = 3.3e−12 at − 1 position under in vivo condition; no significant difference was detected at − 2 position under in vivo condition; *P* value = 3.1e−5 and < 2.2e−16 at − 1 and − 2 positions under *deproteinized* condition). **e** SHAPE reactivity profiles for alternative 3′ss events with the distal 3′ss as the major one. Average SHAPE reactivity profiles of both in vivo (dark purple) and *deproteinized* (light purple) conditions across different 3′ss and the corresponding branch points are shown. Significantly higher SHAPE reactivity only appears at the branch site of the major distal 3′ss rather than the minor proximal 3′ss (Mann-Whitney test, *P* value = 1.2e−3 and 2.8e−4 at branch point under in vivo and *deproteinized* conditions, respectively). **f** SHAPE reactivity profiles for alternative 3′ss events with the proximal 3′ss as the major one. The significantly higher SHAPE reactivity only appears at the branch point of the major proximal 3′ss rather than the minor distal 3′ss (Mann-Whitney test, *P* value = 1.4e−2 and 1.7e−3 at the branch point under in vivo and *deproteinized* conditions, respectively)

In addition to generating RNA structure information of pre-mRNAs, we also calculated the splicing efficiency for each intron to measure the outcome for splicing events (Additional file 1: Figure S8, see details in the “Methods” section). Since most of the introns showed either very high (≥ 90%) or very low (≤ 10%) splicing efficiencies, two groups of splicing events were classified: spliced events (splicing efficiency ≥ 90%, 32,522 spliced events were identified, Additional file 2) and unspliced events (i.e., intron retention, splicing efficiency ≤ 10%, 4056 unspliced events were identified, Additional file 2). We then compared the average SHAPE reactivities between these two groups of splicing events. Although the exon-intron regions of both these two groups shared similar nucleotide compositions (Additional file 1: Figure S9), distinctive SHAPE reactivity profiles were observed between spliced and unspliced groups (Fig. 3a, b). Specifically, we found that in vivo SHAPE reactivities at the − 1 position immediately upstream of 5′ss were notably higher for spliced events compared to unspliced events (Fig. 3a). Similarly, SHAPE reactivities at the − 1 and − 2 positions upstream of 5′ss were significantly higher in spliced events than those in unspliced events for the *deproteinized* condition (Fig. 3b). These findings indicated that the − 1 and − 2 nucleotides upstream of 5′ss tended to be more single-stranded in spliced events compared to unspliced events. To further confirm this profile observed with average SHAPE reactivity, we plotted the SHAPE reactivities across 5′ss for each exon-intron junction in heatmaps (Additional file 1: Figure S10). Significant higher SHAPE reactivities at − 1 and − 2 positions were observed for most of the spliced events while most unspliced ones did not show this pattern. The heatmaps of SHAPE reactivities for each individual exon-intron junction are consistent with the average SHAPE reactivity profile (Fig. 3a, Additional file 1: Figure S10). In addition, we also performed RNA structure folding with and without SHAPE reactivity across the 5′ splice site (5′ss) for both spliced and unspliced events (Additional file 1: Figure S11). Consistent with the SHAPE reactivity profiles, we found that, for SHAPE-constrained structures of the spliced events, the average unpaired probability at the − 1 and − 2 positions was much higher than their neighboring positions, while this phenomenon is absent in the unspliced events (Additional file 1: Figure S11). We further assessed sequence content across 5′ss in both spliced and unspliced events and found no apparent sequence preference between these two groups (Additional file 1: Figure S9). Thus, our results suggested that this distinctive structure signature was associated with splicing events, but not due to sequence preference.

We then assessed RNA structure features for branch sites and 3′ss regions, which are important for 3′ss recognition during splicing [1]. To assess RNA structure features at branch sites, we predicted branch sites using SVM-BPfinder [32]. Higher SHAPE reactivities were observed at branch sites under both in vivo and *deproteinized* conditions for spliced events compared to unspliced events, indicating single-strandedness at branch sites was associated with splicing (Fig. 3a, b). We also assessed the sequence content across branch sites in both spliced and unspliced groups, and no sequence preference was observed (Additional file 1: Figure S9). SHAPE reactivities of regions immediately upstream of dinucleotide AG at 3′ss (from − 7 to − 4 positions) were relatively lower than flanking regions (Fig. 3a, b). However, there was no significant SHAPE reactivity difference between spliced and unspliced events at 3′ss regions, indicating no direct association with splicing. Collectively, both RNA structure features upstream of 5′ss and at the branch site in pre-mRNAs were associated with splicing.

We then explored whether these RNA structure features are also associated with splice site selection in alternative splicing events. Firstly, we identified alternative 5′ss events from genome annotation and selected those pre-mRNAs with two alternative 5′ss (5116 alternative 5′ss events were identified and used in the following analysis, Additional file 3). We then classified the two alternative 5′ss as distal and proximal 5′ss, according to their relative positions. Based on the expression levels of the corresponding isoforms, we then identified the major 5′ss (≥ 80% of total abundance of the two isoforms) and the minor 5′ss (≤ 20% of total abundance of the two isoforms) (see details in the “Methods” section). We found that SHAPE reactivities at the − 1 and − 2 positions upstream of 5′ss were significantly higher in the major 5′ss group than those in the minor 5′ss group, regardless of distal or proximal positions (Fig. 3c, d). Therefore, the two-nucleotide single-stranded RNA structure feature upstream of 5′ss was associated with the selection of alternative 5′ss. We then performed the corresponding assessment for alternative 3′ss events (9237 alternative 3′ss events were identified and used in the following analysis, Additional file 4) and found SHAPE reactivities at branch sites were notably higher in the major 3′ss group compared to the minor 3′ss group, regardless of distal or proximal positions (Fig. 3e, f). Thus, single-strandedness at the branch site was associated with the selection of alternative 3′ss. Taken together, RNA structure features identified upstream of 5′ss and at the branch site were also strongly associated with the recognition of alternative 5′ss and 3′ss in alternative splicing events.

### The two-nucleotide single-stranded RNA structure feature upstream of 5′ss is sufficient to regulate splicing

**Fig. 4:**
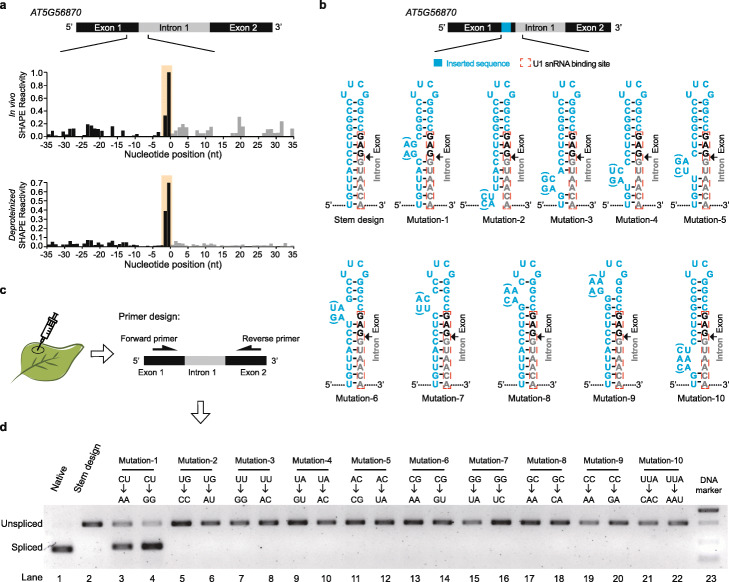
The two-nucleotide single-stranded RNA structure feature at − 1 and − 2 nt positions upstream of 5′ss can regulate splicing. **a** SHAPE reactivity profiles across 5′ss of the first intron of *AT5G56870*. High SHAPE reactivities are observed at − 1 and − 2 nt positions (shaded in yellow) upstream of 5′ss under both in vivo (top) and *deproteinized* (bottom) conditions, which resemble the global SHAPE reactivity profiles for spliced events. **b** Schematic of experimental design to validate the effect of single-strandedness at the − 1 and − 2 positions of 5′ss on splicing of the first intron of *AT5G56870*. A short sequence (blue) was inserted immediately upstream of the U1 snRNA binding site (red dashed box) to form a stable hairpin structure with the whole U1 binding site completely base-paired. The exon and intron sequences are colored in black and gray, respectively. A series of mutations were introduced at different positions of the inserted sequence to disrupt the base-pairing status of different nucleotides. Two types of mutations (with/without bracket) were designed for each position to avoid potential effects due to changing the sequence content. **c**, **d** Determination of splicing events by transient expression assay in *Nicotiana benthamiana*. The spliced and unspliced products were distinguished by semiquantitative RT-PCR using the same pair of primers located upstream and downstream of the intron. Spliced and unspliced products are indicated by bands with different sizes. The construct with native sequence was successfully spliced (lane 1). Splicing was completely inhibited in the stem design (lane 2). The mutation “AA” or “GG” disrupted the base-pairing status at − 1 and − 2 positions upstream of 5′ss (Mutation-1) and rescued the splicing (lanes 3 and 4). All other mutations (Mutation-2–10) designed to disrupt other base-pairing sites across the U1 binding site did not rescue the splicing (lanes 5–22). Lane 23, the DNA marker

A nucleotide with high GC content tends to be more double-stranded [33]. Thus, the distinctive single-strandedness at the − 1 nucleotide upstream of 5′ss, as a conserved G, is unexpected. In addition, the − 1 and − 2 nucleotide positions lie within the nine-nucleotide binding region of U1 snRNA (from − 3 to + 6 nt region of 5′ss) during splicing [34]. If this splicing associated RNA structure feature we observed, affected U1 snRNA binding, then a similar RNA structure feature should have been observed across the whole binding site. However, high SHAPE reactivities were only observed for two out of nine nucleotides rather than the whole binding site. Consequently, we tested whether these two single-stranded nucleotides upstream of 5′ss were sufficient to regulate splicing. We selected the first exon-intron-exon region of *AT5G56870* successfully spliced as a representative example of the pre-mRNAs comprising this distinctive two-single-stranded RNA structure feature upstream of 5′ss (Fig. 4a, Additional file 1: Figure S12). The single-strandedness of − 1 and − 2 positions was further confirmed by RNA structure model constrained with SHAPE reactivity (Additional file 1: Figure S13). We then made use of it for our functional validation. To avoid disrupting base-pairing between 5′ss and U1 snRNA during splicing, we maintained the U1 snRNA binding site sequence content and inserted a short sequence immediately upstream of this U1 binding site to form a stable hairpin structure with the whole U1 binding site completely base-paired (illustrated in Fig. 4b). Then, we introduced a series of mutations in the inserted sequence that base-pair with the U1 binding site in order to disrupt the base-pairing status of different nucleotides (Fig. 4b). We assessed the splicing events on these designed constructs through transient expression assays in *Nicotiana benthamiana* (Fig. 4c). First, we confirmed that the native sequence construct was successfully spliced in tobacco leaves (Fig. 4d, lane 1 of *AT5G56870*). Splicing was completely inhibited when the whole U1 snRNA binding site was completely base-paired with the inserted sequence upstream (Fig. 4d, lane 2 of *AT5G56870*). By introducing a mutation “AA” to allow base-pairing disruption at − 1 and − 2 positions upstream of 5′ss, we found splicing was rescued (Fig. 4d, lane 3 of *AT5G56870*). To avoid potential effects due to changing the sequence content, we also mutated these two nucleotides to “GG” that also disrupted the base-pairing status at − 1 and − 2 positions and found splicing was also rescued (Fig. 4d, lane 4 of *AT5G56870*). Furthermore, we assessed the other mutations designed to disrupt other base-pairing sites across the whole U1 binding site (Fig. 4d, lanes 5–22). Remarkably, structure disruptions of all other base-pairing sites, even a three-nucleotide mutation, were not able to rescue splicing (Fig. 4d, lanes 5–22). We also performed the experimental validation with the same design in two additional genes (*AT1G08450* and *AT3G08930*, Additional file 1: Figure S14). All these results showed that only the mutations disrupting the base-pairing of − 1 and − 2 positions can significantly restore splicing (Fig. 4d, Additional file 1: Figure S14). Hence, our results indicated that only the two-nucleotide single-stranded RNA structure feature at − 1 and − 2 positions upstream of 5′ss was sufficient to regulate splicing.

### A unique RNA structure feature on pre-mRNAs is associated with polyadenylation and alternative polyadenylation

Another key step of mRNA processing is polyadenylation that starts with endonucleolytic cleavage on pre-mRNAs followed by addition of a poly(A) tail at the cleavage site [2]. Since only the pre-mRNA structure before endonucleolytic cleavage can be used for elucidating the mechanism underpinning polyadenylation, we assessed whether pre-mRNAs before endonucleolytic cleavage were enriched in our Nuc-SHAPE-Structure-Seq libraries. We compared the sequencing read coverage across cleavage sites (poly(A) sites) annotated in a previous study [35] with both our Nuc-SHAPE-Structure-Seq libraries and cytosolic SHAPE-Structure-Seq libraries. The reads across poly(A) sites were highly enriched in our Nuc-SHAPE-Structure-Seq libraries compared to our cytosolic SHAPE-Structure-Seq data (Additional file 1: Figure S15). This indicated high enrichment of pre-mRNAs before polyadenylation in our Nuc-SHAPE-Structure-Seq libraries (Additional file 1: Figure S15).

**Fig. 5:**
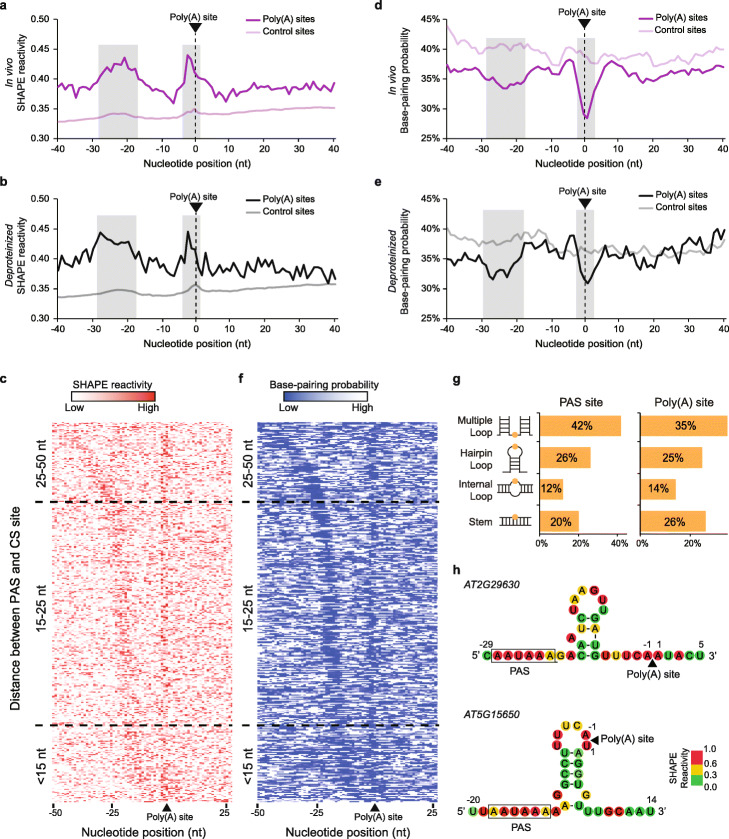
Two single-stranded regions on pre-mRNA are associated with polyadenylation. **a**, **b** SHAPE reactivity profiles across poly(A) sites for in vivo (**a**) and *deproteinized* (**b**) conditions. The *X*-axis represents the relative position to the poly(A) site. Average SHAPE reactivities in two regions (gray shaded) were significantly higher compared to flanking regions for both in vivo (purple) and *deproteinized* (black) conditions (Fisher’s exact test, *P* value = 3.1e−14 and 3.6e−6 for in vivo; *P* values = 4.7e−12 and 1.4e−3 for *deproteinized*). The numbers of control sites are 4575 and 983 for in vivo and *deproteinized* conditions, respectively. The numbers of poly(A) sites are 3077 and 551 for in vivo and *deproteinized* conditions, respectively. **c** Heatmap showing in vivo SHAPE reactivity profiles across the PAS motif “AAUAAA” and poly(A) site. The pre-mRNAs are sorted by the distance between PAS and poly(A) site. The gradient color from light to dark red represents SHAPE reactivity from low to high. The SHAPE reactivities are much higher at both the PAS and poly(A) sites compared to flanking regions. **d**, **e** Base-pairing probability (BPP) profiles across poly(A) sites for in vivo (**d**) and *deproteinized* (**e**) conditions. Average BPPs in two regions (gray shaded) were significantly lower compared to flanking regions for both in vivo (purple) and *deproteinized* (black) conditions (Fisher’s exact test, *P* value = 5.0e−12 and 1.4e−7 for in vivo; *P* values = 1.3e−8 and 2.8e−6 for *deproteinized*). **f** Heatmap showing in vivo BPPs across the conventional PAS motif “AAUAAA” and poly(A) site. **g** Classification of RNA structure elements across the PAS and poly(A) sites. The three different single-stranded types (multiple loop, hairpin loop, and internal loop) and the double-stranded stem type were assessed for all the PAS and poly(A) sites. The percentage of each type is shown. Most of the PAS and poly(A) sites are located in the single-stranded loop regions. **h** Illustrations of two individual pre-mRNA structures with both the PAS and poly(A) sites located in single-stranded loop regions. Nucleotides were color-coded according to the in vivo SHAPE reactivity values

To accurately determine RNA structure features across poly(A) sites, only reads mapped across poly(A) sites and in downstream flanking regions were used to generate SHAPE reactivity profiles (3077 and 551 poly(A) sites with ≥ 1 RT-stop per nucleotide under in vivo and *deproteinized* conditions were used in the analysis, Additional files 5, 6). We found that average SHAPE reactivities in two regions (from − 28 to − 17 nt upstream of the poly(A) site and from − 4 to + 1 nt across the poly(A) site) were significantly higher compared to flanking regions for both in vivo and *deproteinized* conditions (Fig. 5a, b), suggesting these two regions tended to be more single-stranded than flanking regions. To eliminate the effect of nucleotide composition, we identified control sites where nucleotide composition was similar to the sequence content across poly(A) sites, but where polyadenylation did not occur (Additional file 1: Figure S16). We found no significant RNA structure features across these control sites, indicating the two single-stranded regions observed across the poly(A) sites above were specifically associated with polyadenylation (Fig. 5a, b). Furthermore, we assessed whether these two single-stranded regions also appeared in alternative polyadenylation sites. Compared to constitutive poly(A) sites, we found a similar but weaker structure feature across alternative polyadenylation sites (Additional file 1: Figure S17, Additional files 7, 8). Notably, these structure features were different to those identified from a previous RNA structurome study on mature mRNAs [24], further indicating structure differences between pre-mRNAs and mature mRNAs. Therefore, this RNA structure feature with two single-stranded regions may also be responsible for alternative polyadenylation.

Further investigation of the sequence content in positions − 28 to − 17 nt upstream of poly(A) sites showed that this region had an accumulation of the conventional polyadenylation signal (PAS) motif “AAUAAA” (Additional file 1: Figure S18, Additional file 9). We then aligned SHAPE reactivities across this conventional PAS motif “AAUAAA” upstream of poly(A) sites and sorted pre-mRNAs by the distance between PAS and poly(A) sites (Fig. 5c). The corresponding SHAPE reactivities across PAS and poly(A) sites for each pre-mRNA were then plotted as a heatmap (Fig. 5c). We found that SHAPE reactivities were higher at both PAS sites and across poly(A) sites compared to flanking regions (Fig. 5c). Thus, the conventional polyadenylation signal (PAS) motif “AAUAAA” tended to be a single-stranded region. Interestingly, this unique structure feature consistently appeared regardless of the distance between PAS and poly(A) sites (Fig. 5c). Hence, our results suggested that the single-strandedness of both PAS and poly(A) sites may serve as RNA structure signals for polyadenylation.

To understand what type of RNA structures could be formed with these two single-stranded regions, we folded sequences across the poly(A) sites with the constraints of SHAPE reactivities by using the *Vienna RNAfold* package [36]. We then calculated the base-pairing probability (BPP) of each nucleotide [36]. Consistent with our SHAPE reactivity profiles, we found that the BPPs in these two regions (from − 28 to − 17 nt upstream of the poly(A) site and from − 4 to + 1 nt across the poly(A) site) were significantly lower compared to the flanking regions for both in vivo and *deproteinized* conditions, confirming the single-strandedness of these two regions (Fig. 5d, e). Furthermore, we found no obvious BPP features across the control sites, indicating this structure feature was not due to preferential nucleotide composition (Fig. 5d, e). We also generated the heatmap of BPPs across the conventional PAS motif “AAUAAA” and poly(A) sites. We found that the BPPs were much lower at both PAS sites and poly(A) sites compared to flanking regions (Fig. 5f), consistent with SHAPE reactivity profiles (Fig. 5c). In addition, we assessed the detailed RNA structure elements across PAS and poly(A) sites using the *Forgi* utility [37]. We found that most RNA structures had both PAS and poly(A) sites located in single-stranded loop regions including multiple loop, hairpin loop, and internal loop (Fig. 5g). For instance, one type of RNA structure comprised both PAS and poly(A) sites located in multiple loop regions and connected by one hairpin structure (an example is illustrated in Fig. 5h—top). Another type of RNA structure comprised the PAS site located in a multiple loop region with the poly(A) site located in a hairpin loop region (an example is illustrated in Fig. 5h—bottom). Therefore, our results indicated that diverse RNA structures were formed to maintain single-strandedness at both PAS and poly(A) sites.

## Discussion

For the first time, we generated the in vivo RNA structure landscape of *Arabidopsis* nuclear RNAs with structure information for all four nucleotides by developing Nuc-SHAPE-Structure-Seq. Having achieved high coverage and high accuracy with our Nuc-SHAPE-Structure-Seq, we were able to investigate global RNA structure features of nuclear mRNAs and uncover the regulatory role of RNA structure in mRNA processing.

### Nuclear mRNAs fold differently from cytosolic mRNAs across translation start and stop sites

Cytosolic mRNAs are the processed products from nuclear mRNAs; thus, they share the same sequences. An intriguing question is whether nuclear mRNA structures in these regions are the same as cytosolic mRNA structures? Here, we found that RNA structure features downstream of start codons and at stop codons were significantly different between nuclear and cytosolic mRNAs (Fig. 2). A previous in vitro study suggested that mature mRNAs might require strong structures downstream of the start codon for increasing the 40S subunit “dwell time” [38]. Our observation (Fig. 2a) implied that stronger structures downstream of the start codon in cytosolic mRNAs compared to nuclear mRNAs might relate to the ribosome pausing in vivo. At stop codons, we found much higher SHAPE reactivities in cytosolic mRNAs (Fig. 2b). This single-stranded structure feature was also observed in a previous RNA structurome study and was suggested to facilitate translation termination [39]. But in nuclear mRNAs, this structure feature was much weaker (Fig. 2b), implying this single-stranded structure feature at stop codons in cytosolic mRNAs might be specific for translation termination. Taken together, these structure feature differences between nuclear and cytosolic mRNAs implied that mRNAs might undergo refolding from the nucleus to the cytosol.

In addition to the effects on structure differences from translation, mRNA processing, e.g., polyadenylation and splicing, might also impact the folding status of RNA structures in different cellular compartments. Previous RNA structure profiling of mature mRNAs after polyadenylation in human observed more folded structure features in the region downstream of PAS sites compared to the region upstream of PAS, which were found to facilitate polyadenylation [15]. However, we did not observe significant structure differences between these two regions in our Nuc-SHAPE-Structure-Seq, suggesting mRNAs might be refolded after polyadenylation (Fig. 5a, b). In addition, we found a distinctive single-stranded region across poly(A) sites (Fig. 5a, b), demonstrating that our method had overcome the limitations of previous mature mRNA structurome studies, which lacked structure information across poly(A) sites [15]. Furthermore, our previous study on mature mRNAs in *Arabidopsis* revealed that significantly more folded structure features formed upstream of alternative polyadenylation sites compared to flanking regions [24]. However, we found RNA structure features associated with alternative polyadenylation in the pre-mRNAs before polyadenylation (Additional file 1: Figure S17) were different from those observed in mature mRNAs [24]. Additionally, our previous study on mature RNAs showed a stronger RNA structure feature upstream of 5′ss in unspliced events [24]. However, we did not observe similar features in our Nuc-SHAPE-Structure-Seq (Fig. 3a, b), indicating the RNA structure features related to splicing are also different between pre-mRNAs and mature mRNAs [24]. Thus, these structure differences before and after mRNA processing implied that mRNAs may adopt different structures for serving distinct biological processes. Many other factors, e.g., diverse protein interactions, RNA modifications, and distinct cellular conditions between the nucleus and cytosol, may also contribute to these structure differences, which offers scope for future studies.

### Distinctive RNA structure features upstream of 5′ss and at the branch site are associated with recognizing 5′ss and 3′ss, respectively

Previous in vitro enzymatic RNA structure profiling in *Arabidopsis* nuclear RNAs showed greater structure differences at the exon-intron junctions where the 5′ end of introns was much more double-stranded than upstream exons and 3′ end of introns was more single-stranded than flanking sequences [14]. However, we did not observe these dramatic differences across exons and introns in our Nuc-SHAPE-Structure-Seq data, further confirming that in vivo RNA structures were different from in vitro RNA structures [19, 20]. The recognition of both 5′ss and 3′ss is of great importance during splicing [1, 4]. The consensus sequence motifs for splice sites are so short that a large number of sites with matching sequences are widely spread in the transcriptome [4]. How to distinguish actual splice sites from a large number of false positives has been a primary challenge in splice site recognition [4]. Previous individual studies in human suggested strong RNA structures at U1 and U2 snRNA binding sites can prevent the interactions with U1 and U2 snRNA, thus interfering with the recruitment of U1 and U2 snRNPs during splicing [40–42]. In our transcriptome-wide analysis for 5′ss, we identified a two-nucleotide single-stranded RNA structure feature immediately upstream of the 5′ss, which was associated with splicing events (Fig. 3a, b, Additional File 1: Figure S10). Since the structure feature was located within the U1 snRNA binding region (from − 3 to + 6 position across the 5′ss) [34], it is likely that the single-strandedness of these two nucleotides promotes the binding of U1 snRNA in 5′ss recognition. For 3′ss, we found the single-strandedness at the branch site was associated with splicing events (Fig. 3a, b). Since U2 snRNA binds across the branch point through base-pairing [1], the single-strandedness at the branch site might promote the binding of U2 snRNA in 3′ss recognition. Alternatively, this single-strandedness might also be a consequence after binding with U2 snRNA since the RNA-RNA base-pairing interaction leaves the branch point as an internal bulge [1]. Previous studies in yeast suggested that stem-loop structures between the branch site and 3′ss could promote the recognition of 3′ss [43, 44]. We also found a 4-nt low SHAPE reactivity region upstream of AG dinucleotides at the 3′ss, which suggested the formation of a stronger RNA structure between 3′ss and the branch site (Fig. 3a, b). However, this structure feature was not associated with splicing events and, as such, might be linked with subsequent steps after the recognition of 3′ss, such as docking the 3′ss into the reaction center to approach 5′ss [45]. Notably, the two-nucleotide single-stranded RNA structure feature upstream of 5′ss and the single-strandedness at the branch point were also strongly associated with the selection of alternative 5′ss and 3′ss, respectively (Fig. 3c–f). These results further suggested that these two in vivo RNA structure features might serve as general rules for determining actual 5′ss and 3′ss in splicing.

### The two-nucleotide single-stranded RNA structure feature upstream of 5′ss can regulate splicing

Previous studies of individual RNA structure suggested that strong RNA structures formed at 5′ss can inhibit U1 snRNA binding, and subsequently repress splicing [8, 41, 42]. However, the strong structures in each case were so different that no general RNA structure features have been identified for regulating splicing. From our Nuc-SHAPE-Structure-Seq data, we were able to sensitively determine that a very fine RNA structure feature showing single-strandedness at the − 1 and − 2 positions upstream of 5′ss was associated with splicing at the transcriptome-wide scale (Fig. 3a, b). Our functional assessment further confirmed that fine-tuning RNA structure by switching the base-pairing status of only these − 1 and − 2 positions upstream of 5′ss was sufficient to change the fate of splicing (Fig. 4, Additional file 1: Figure S14).

One possible mechanism is the single-strandedness of the − 1 and − 2 positions upstream of 5′ss promoted splicing by facilitating the binding of U1 snRNA. U1 snRNA base-pairs with a total of nine nucleotides (from − 3 to + 6 region of 5′ss) across 5′ss [34]. Thus, any nucleotides within this nine-nucleotide U1 binding site should have been able to affect splicing. However, we observed that single-strandedness at all other nucleotide positions within the U1 binding site (except for the − 1 and − 2 positions) was not able to rescue splicing events (Fig. 4b, d). Therefore, our study revealed that the position of this two-nucleotide single-stranded RNA structure feature was also important for regulating splicing. This phenomenon raised the possibility that the − 1 and − 2 nucleotides upstream of 5′ss may be the first positions for the interaction with U1 snRNA. Further biophysics studies might be able to assess this hypothesis. Furthermore, once the 5′ss is recognized by base-pairing with U1 snRNA, the spliceosome is assembled onto the intron region and the 5′ss-U1 interaction is replaced by interactions of 5′ss with U5 snRNA (from − 3 to − 1 region of 5′ss) [46]. It is possible that the single-strandedness of the − 1 and − 2 positions may also promote interaction with U5 snRNA. Taken together, both our transcriptome-wide RNA structure profiling and functional assessment indicated that the two-nucleotide single-stranded structure feature at the − 1 and − 2 positions upstream of 5′ss can serve as a general role in splicing regulation.

Since splicing is a fundamental biological process across eukaryotes, the regulatory motif for splicing is likely to be conserved and highly selected during evolution. Previous identification of the most conserved sequence motif required for 5′ss recognition is as short as only a dinucleotide GU at 5′ss [4]. The sequence requirement of only two nucleotides might be minimized during evolution selection. The short sequence length of the conserved nucleotides might provide the plasticity for flanking nucleotides to contribute to other biological functions. Here, we postulate that the very fine RNA structure feature we identified from the transcriptome is likely to have evolved in a similar way as the sequence motif, in terms of the single-strandedness of only two nucleotides being sufficient to regulate splicing. It will be of great interest to extend our study in other species to investigate the generality of this regulatory mechanism.

### Two single-stranded regions upstream and across poly(A) sites are associated with both polyadenylation and alternative polyadenylation

Similar to the challenge of how to recognize splice sites, the recognition of poly(A) sites does not always rely on sequence content. In particular, no unique sequence motif exists around poly(A) sites in plants [11, 47]. Indeed, only ~ 10% of *Arabidopsis* genes contain the conventional PAS motif “AAUAAA” upstream of poly(A) sites [11]. Therefore, how to precisely determine actual poly(A) sites has been a major question for improving our understanding of polyadenylation regulation. A previous enzymatic probing study on in vitro nuclear RNAs in *Arabidopsis* had attempted to investigate RNA structure features at poly(A) sites [14]. However, no structure features were observed at either polyadenylation or alternative polyadenylation sites [14], which may be due to the low resolution of enzymatic probing [6, 14]. Here, we identified two single-stranded regions (from − 28 to − 17 nt upstream of the poly(A) sites and from − 4 to + 1 nt across the poly(A) sites) that were associated with both polyadenylation and alternative polyadenylation (Fig. 5a, b, d, e, Additional file 1: Figure S17). These RNA structure features did not appear in the regions where the nucleotide composition was similar but polyadenylation did not occur (Fig. 5a, b, d, e). Hence, these close-by two single-stranded RNA structure features may serve as an additional signature for the recognition of poly(A) sites. We also observed that the overall SHAPE reactivities in control sites were lower than those in true poly(A) sites, which suggested that the 3′ end of nascent mRNA before polyadenylation tends to be more accessible than other genic regions in living cells (Fig. 5a, b). This is consistent with the previous observation that single-stranded RNA features are required for recruitment of 3′ processing machinery [48].

Interestingly, most conventional PAS motifs “AAUAAA” are located within the region from − 28 to − 17 nt upstream of the poly(A) sites (Additional file 1: Figure S18). We did observe the conventional PAS motif “AAUAAA” region was more single-stranded compared to flanking regions (Fig. 5c, f), which suggested that the single-stranded region upstream of the poly(A) site corresponded to the PAS motif site. Since sequence content is insufficient for predicting PAS sites [11], the single-stranded region upstream of poly(A) sites could offer another signature for recognizing the unconventional PAS motif. Moreover, the interactions of the PAS sites with CPSF30 and WDR33 proteins are crucial during polyadenylation [2]. Hence, PAS sites might adopt this single-stranded structure feature to facilitate protein binding. Furthermore, the endonucleolytic cleavage at poly(A) sites is catalyzed by CPSF73, which has been suggested to prefer RNA single-strandedness [49]. Therefore, the single-stranded region across poly(A) sites might facilitate the interaction between CPSF73 and poly(A) sites.

## Conclusions

In summary, we generated the in vivo nuclear RNA structure landscape in *Arabidopsis* achieving both high resolution and accuracy with our Nuc-SHAPE-Structure-Seq method. We revealed the structural differences between nuclear and cytosolic mRNAs. We successfully identified respective pre-mRNA structure features associated with splicing and polyadenylation. Through functional validation, we determined an RNA structure feature which can regulate splicing. Our study unveiled a new RNA structure regulatory mechanism for mRNA processing. Also, our work emphasized the importance of dissecting RNA populations from different stages of the mRNA life cycle in order to investigate the relationship between RNA structure and biological functions.

## Methods

### Plant materials and growth condition

*Arabidopsis thaliana* (Col-0) seeds were sterilized with 70% (v/v) ethanol and plated on half-strength Murashige and Skoog medium (1/2 MS). The plates were wrapped in foil and stratified at 4 °C for 3 days and then grown in a growth chamber at 22 °C for 5 days.

### Nuclei isolation and quality control

Nuclei isolation from 5-day-old *Arabidopsis* seedlings was performed according to the previous protocol [50], which assured the isolation of high quality, intact nuclei. Briefly, *Arabidopsis* seedlings were chopped in the nuclei isolation buffer to release nucleus. The nuclei-containing solution was then filtered through nylon mesh filter, and the nuclei pellet was collected by centrifugation. The supernatant was retained as the cytosolic fraction [50]. Nuclear RNAs and cytosolic RNAs were extracted separately from the pellet and the supernatant by using RNeasy Plant Mini Kit (Qiagen). The intactness of isolated nuclei was examined under fluorescent microscopy after DAPI (4′,6-diamidino-2-phenylindole) staining. The purity of nuclei was confirmed by western blot of histone H3 and cytoplasmic marker protein PEPC (phosphoenolpyruvate carboxylase) [14]. Western blots using proteins extracted from the purified nucleus, the cytosolic supernatant fraction, and the whole cells were performed with anti-histone H3 (Sigma) and anti-PEPC (Agrisera) antibodies.

### In vivo and *deproteinized* RNA structure probing

For in vivo RNA structure probing, *Arabidopsis* seedlings were completely covered in 20 ml SHAPE reaction buffer (100 mM KCl, 40 mM HEPES (pH 7.5), and 0.5 mM MgCl_2_) in 50 ml Falcon tubes. The NAI (2-methylnicotinic acid imidazolide) treatment was performed with a final concentration of 100 mM as previously reported [22]. The (−)SHAPE treatment was performed by adding the same amount of anhydrous DMSO. The reaction was performed at 22 °C for 15 min [22]. Freshly prepared DTT (dithiothreitol) was added to a final concentration of 0.5 M to quench the reaction [22]. The seedlings were then used for nuclei isolation and RNA extraction performed as described above. For *deproteinized* RNA structure probing, *Arabidopsis* nuclei were lysed in lysis buffer (50 mM Tris-HCI pH 8.0, 10 mM EDTA pH 8.0, 1% SDS). The lysate was deproteinized by passing through two phenol followed by one chloroform extractions [29]. Then, RNAs were subjected to NAI treatment immediately at 22 °C for 15 min followed by DTT quenching, Micro Bio-Spin P6 column (Bio-Rad) purification and RNA extraction as described above [22].

### SHAPE-Structure-Seq library construction

SHAPE-Structure-Seq library construction was followed and modified according to previous methods [22, 51]. The genomic DNA was removed after RNA extraction using Turbo DNase Kit (Ambion). Ribosomal RNA (rRNA) depletion was performed by using ribo-zero magnetic kit (Illumina). The rRNA-depleted RNAs were purified and recovered by using RNA clean and concentrator (Zymo research) after RNA fragmentation [25]. 3′ dephosphorylation was performed by using T4 PNK enzyme (NEB) at 37 °C for 30 min. Next, 3′ adaptor (5′-/5rApp/AGATCGGAAGAGCACACGTCTG/3SpC3/-3′) was ligated to the RNAs at 25 °C for 1 h using T4 RNA ligase 2 (NEB). The RNAs were then subjected to reverse transcription using SuperScript III (Thermo Fisher Scientific) with reverse primer (5′-CAGACGTGTGCTCTTCCGATCT-3′). The synthesized first-strand cDNAs were purified by 10% TBE-Urea Gel (Thermo Fisher Scientific) followed by gel purification to enrich cDNA fragments from SHAPE modified RNA by size selection (Additional file 1: Figure S2). Next, the 5′ adaptor ligation was performed by ligating the adaptor (5′-5Phos/AGATCGGAAGAGCGTCGTGTAGCTCTTCCGATCTNNNNNN/3SpC3-3′) to the purified cDNA using Quick T4 DNA ligase (NEB) at 20 °C overnight. The ligated cDNAs were then purified by 10% TBE-Urea Gel (Thermo Fisher Scientific). The PCR reaction was performed using forward primer (5′-AATGATACGGCGACCACCGAGATCTACACTCTTTCCCTACACGACGCTCTTCCGATCT-3′) and reverse primer (e.g., index 2) (5′-CAAG CAGAAGACGGCATACGAGATACATCGGTGACTGGAGTTCAGACGTGTGCTCTTCCGATCT-3′, Additional file 1, Table S3) with 2× HiFi readymix (KAPA) followed by agarose gel purification. The libraries were then subjected to 100 nt paired-end sequencing on Illumina HiSeq 4000 by Beijing Genomics Institute, Shenzhen, China.

### Transient expression assay in *Nicotiana benthamiana*

The sequences of the exon-intron-exon from *AT5G56870*, *AT1G08450*, and *AT3G08930* were cloned into expression vector inter2 using Gibson Assembly system (NEB) with designed primers (Additional file 1: Table S3). Corresponding mutations were introduced using Q5 Site-Directed Mutagenesis kit (NEB) with designed primers (Additional file 1: Table S3) for *AT5G56870*. For *AT1G08450* and *AT3G08930*, the exon-intron-exon sequence with designed mutations was synthesized (Sangon Biotech (Shanghai) Co., Ltd) and cloned into expression vector inter2. All constructs were transformed into *Agrobacterium tumefaciens* strain GV3101 by electroporation. Transient expression was carried out in *Nicotiana benthamiana* according to the previous protocol [52]*.* RNA was extracted with RNeasy Plant Mini Kit (Qiagen), and semiquantitative RT-PCR was performed with designed primers (Additional file 1: Table S3).

### Reads mapping and SHAPE reactivity calculation

The quality of SHAPE-Structure-Seq raw reads was assessed using FastQC (Ver. 0.11.5), and the low-quality reads were filtered out. Adapter trimming was performed using Cutadapt (Ver.1.14). *Arabidopsis* genome reference sequences were obtained from TAIR10 database (https://www.arabidopsis.org/). The genome index was built by Hisat2-build with GTF file containing the annotation of additional alternative splicing isoforms from AtRTD2 database [26]. Sequencing reads were aligned onto genome sequence by using Hisat2 (Ver.2.1.0) with “--no-softclip, --no-mix, --reorder, -k 10” option. After the reads mapping, only uniquely mapped reads were retained for the following analysis. Reverse-transcription stops (RT-stops) were counted followed by SHAPE reactivity calculation and normalization according to the previous method [53].

### Splicing associated pre-mRNA structure analysis

We first filtered out split read alignments and only retained reads mapped to genome sequences in unsplit manners. Then, to precisely reflect the RNA secondary structure of pre-mRNA before splicing, only reads mapped to exon-intron junction and intron regions (i.e., those reads from transcripts before intron removal) were used for RT-stop counting and SHAPE reactivity calculations. SHAPE reactivity values were normalized to 0–1 according to the previous method [53]. When a read is mapped to the reference genome, the actual structure stop is on the nucleotide that is 1 nt upstream to the 5′ end of the mapped read. Therefore, the reads mapped immediately downstream of 3′ss cannot be confidently assigned to either the upstream 5′ exon (spliced isoform) or the upstream intron (unspliced isoform). Thus, the last two dinucleotides at 3′ss were excluded from the SHAPE reactivity calculation. The branch site in intron was predicted by SVM-BPfinder [32].

The (−)SHAPE Structure-seq of cytosolic mRNAs was used for calculating the splicing efficiency of each intron. Splicing efficiency was calculated by the formula:

$$ \mathrm{Splicing}\ \mathrm{Efficiency}=\frac{\mathrm{Spliced}\ \mathrm{Mapped}\ \mathrm{Reads}}{\mathrm{Spliced}\ \mathrm{Mapped}\ \mathrm{Reads}+\frac{\mathrm{Reads}\ \mathrm{across}\ {5}^{\prime}\mathrm{ss}+\mathrm{Reads}\ \mathrm{across}\ {3}^{\prime}\mathrm{ss}}{2}} $$

as shown in Additional file 1: Figure S8a [54]. The spliced and unspliced events were defined according to the splicin*g* efficiency ≥ 90% and ≤ 10%, respectively (Additional file 1: Figure S8b).

For alternative 5′ss analysis, genes with two alternative 5′ss were identified based on the genome annotation (GTF) file from AtRTD2 database [26]. These two alternative 5′ss were then classified as distal and proximal 5′ss according to their relative positions. Then, split mapping reads supporting these two 5′ss were counted and used to represent the expression abundance of corresponding alternative splicing isoforms. If the expression abundance of one alternative splicing isoform accounted for more than 80% of the total abundance of these two splicing isoforms, the corresponding 5′ss was defined as the major 5′ss, and the other as the minor 5′ss accordingly.

For alternative 3′ss analysis, genes with two alternative 3′ss were identified based on the genome annotation (GTF) file from AtRTD2 database [26]. These two alternative 3′ss were then classified as distal and proximal 3′ss according to their relative positions. Then, split mapping reads supporting these two 3′ss were counted and used to represent the expression abundance of corresponding alternative splicing isoforms. If the expression abundance of one alternative splicing isoform accounted for more than 80% of the total abundance of these two splicing isoforms, the corresponding 3′ss was defined as the major 3′ss, and the other as the minor 3′ss accordingly.

### Polyadenylation associated pre-mRNA structure analysis

Poly(A) site annotation for each pre-mRNA was sourced from the previous study [35]. To obtain RNA structure information of pre-mRNAs before polyadenylation, only reads mapped across or downstream of poly(A) sites were used for RT-stop counting and SHAPE reactivities calculated as described above. To select nucleotide composition control sites for poly(A) sites, the occurrence rate of the four nucleotides (A, U, C, and G) at each position across the 40 nt upstream and downstream of true poly(A) sites was used as the reference for identifying the control sites in the transcriptome that were not located at true poly(A) sites.

RNA structure prediction with the constraints of SHAPE reactivity was performed by *RNAfold* in the *Vienna* package (Ver.2.4.3) [36]. Then, the base-pairing probability was derived from the corresponding RNA structure ensemble file “_dp.ps.” The RNA structure element assessments for PAS and poly(A) sites were conducted by *Forgi* (Ver.2.0) [36].

## Supplementary Information

**Additional file 1: Figure S1.** Assessment of nucleus isolation. **Figure S2.** The step-by-step procedure of SHAPE-Structure-Seq library construction. **Figure S3.** Summary of SHAPE-Structure-Seq libraries. **Figure S4.** High reads coverages of the nuclear SHAPE-Structure-Seq libraries. **Figure S5.** Average Pearson correlation coefficient (PCC) of SHAPE reactivities between the two biological replicates of the in vivo nuclear and cytosolic SHAPE-Structure-Seq libraries for mRNAs with different RT-stop reads counts. **Figure S6.** Comparison of SHAPE reactivity profiles with previously published mRNA secondary structure models. **Figure S7.** The high enrichment of pre-mRNAs in the nuclear SHAPE-Structure-Seq libraries. **Figure S8.** The formula for calculating the splicing efficiency and the identification of spliced and unspliced events. **Figure S9.** Similar nucleotide composition between spliced and unspliced events. **Figure S10.** Heatmaps showing the SHAPE reactivities across 5’ss for spliced and unspliced events. **Figure S11.** SHAPE reactivity and unpaired probability profiles of the two biological replicates across 5’ splice site. **Figure S12.** The SHAPE reactivity values across the 5’ss of the first intron of *AT5G56870.* **Figure S13.** Minimum free energy RNA structure at the 5’ss of *AT5G56780* intron 1 folded by *RNAfold*. **Figure S14.** The two-nucleotide single-stranded RNA structure feature at -1 and -2 nt positions upstream of 5’ss can regulate splicing on the genes *AT1G08450* and *AT3G08930*. **Figure S15.** Comparison of reads coverage across poly(A) site between nuclear and cytosolic mRNA libraries. **Figure S16.** Similar nucleotides composition between the poly(A) sites and the control sites. **Figure S17.** A similar but weaker structure feature across the alternative polyadenylation sites. **Figure S18.** An accumulation of the conventional polyadenylation signal (PAS) motif “AAUAAA” in the -28 nt to -17 nt upstream of the poly(A) sites. **Table S1.** Summary of reads mapping for each library. **Table S2.** Comparison between SHAPE reactivity profiles and phylogenetic U1 and U12 snRNA structures. **Table S3.** Summary of primer sequences.

**Additional file 2.** A detailed list of spliced and unspliced introns.

**Additional file 3.** A detailed list of alternative 5' splice site events.

**Additional file 4.** A detailed list of alternative 3' splice site events.

**Additional file 5.** A detailed list of poly(A) sites with high coverage (more than 1 RT-stop per nucleotide) in in vivo libraries.

**Additional file 6. **A detailed list of poly(A) sites with high coverage (more than 1 RT-stop per nucleotide) in *deproteinized* libraries.

**Additional file 7.** A detailed list of alternative poly(A) sites with high coverage (more than 1 RT-stop per nucleotide) in in vivo libraries.

**Additional file 8. **A detailed list of alternative poly(A) sites with high coverage (more than 1 RT-stop per nucleotide) in *deproteinized* libraries.

**Additional file 9.** The position information of Poly(A) signal motif AAUAAA.

**Additional file 10.** Review history.

## Data Availability

Sequence data from this study can be found in the National Center for Biotechnology Information Sequence Read Archive (https://www.ncbi.nlm.nih.gov/sra) under SRA accession SRP214989 [55] of BioProject PRJNA542495 and Gene Expression Omnibus (GEO) accessible through GEO Series accession number GSE135711 [56].
