# Supplementary material for: In vivo nuclear RNA structurome reveals RNA-structure regulation of mRNA processing in plants
Source: Genome Biol. 2021 Jan 4;22:11. doi: 10.1186/s13059-020-02236-4 (PMC7784297; doi:10.1186/s13059-020-02236-4)
Supplement: Supplementary file 1 — Additional file 1: Figure S1. Assessment of nucleus isolation. Figure S2. The step-by-step procedure of SHAPE-Structure-Seq library construction. Figure S3. Summary of SHAPE-Structure-Seq libraries. Figure S4. High reads coverages of the nuclear SHAPE-Structure-Seq libraries. Figure S5. Average Pearson correlation coefficient (PCC) of SHAPE reactivities between the two biological replicates of the in vivo nuclear and cytosolic SHAPE-Structure-Seq libraries for mRNAs with different RT-stop reads counts. Figure S6. Comparison of SHAPE reactivity profiles with previously published mRNA secondary structure models. Figure S7. The high enrichment of pre-mRNAs in the nuclear SHAPE-Structure-Seq libraries. Figure S8. The formula for calculating the splicing efficiency and the identification of spliced and unspliced events. Figure S9. Similar nucleotide composition between spliced and unspliced events. Figure S10. Heatmaps showing the SHAPE reactivities across 5’ss for spliced and unspliced events. Figure S11. SHAPE reactivity and unpaired probability profiles of the two biological replicates across 5’ splice site. Figure S12. The SHAPE reactivity values across the 5’ss of the first intron of AT5G56870. Figure S13. Minimum free energy RNA structure at the 5’ss of AT5G56780 intron 1 folded by RNAfold. Figure S14. The two-nucleotide single-stranded RNA structure feature at -1 and -2 nt positions upstream of 5’ss can regulate splicing on the genes AT1G08450 and AT3G08930. Figure S15. Comparison of reads coverage across poly(A) site between nuclear and cytosolic mRNA libraries. Figure S16. Similar nucleotides composition between the poly(A) sites and the control sites. Figure S17. A similar but weaker structure feature across the alternative polyadenylation sites. Figure S18. An accumulation of the conventional polyadenylation signal (PAS) motif “AAUAAA” in the -28 nt to -17 nt upstream of the poly(A) sites. Table S1. Summary of reads mapping for each library. Table S2. Comparis [file 13059_2020_2236_MOESM1_ESM.pdf]

Additional File 1: Supplementary figures for

In vivo nuclear RNA structurome reveals RNA-structure regulation of mRNA processing in plants

Zhenshan Liu, Qi Liu, Xiaofei Yang, Yueying Zhang, Matthew Norris, Xiaoxi Chen, Jitender Cheema, Huakun Zhang, Yiliang Ding

File list:

Figure S1. Assessment of nucleus isolation.
Figure S2. The step-by-step procedure of SHAPE-Structure-Seq library construction.
Figure S3. Summary of SHAPE-Structure-Seq libraries.
Figure S4. High reads coverages of the nuclear SHAPE-Structure-Seq libraries.
Figure S5. Average Pearson correlation coefficient (PCC) of SHAPE reactivities between the two biological replicates of the *in vivo* nuclear and cytosolic SHAPE-Structure-Seq libraries for mRNAs with different RT-stop reads counts.
Figure S6. Comparison of SHAPE reactivity profiles with previously published mRNA secondary structure models.
Figure S7. The high enrichment of pre-mRNAs in the nuclear SHAPE-Structure-Seq libraries.
Figure S8. The formula for calculating the splicing efficiency and the identification of spliced and unspliced events.
Figure S9. Similar nucleotide composition between spliced and unspliced events.
Figure S10. Heatmaps showing the SHAPE reactivities across 5'ss for spliced and unspliced events.
Figure S11. SHAPE reactivity and unpaired probability profiles of the two biological replicates across 5' splice site.
Figure S12. The SHAPE reactivity values across the 5'ss of the first intron of *AT5G56870*
Figure S13. Minimum free energy RNA structure at the 5'ss of *AT5G56780* intron 1 folded by *RNAfold*.
Figure S14. The two-nucleotide single-stranded RNA structure feature at -1 and -2 nt positions upstream of 5'ss can regulate splicing on the genes *AT1G08450* and *AT3G08930*.
Figure S15. Comparison of reads coverage across poly(A) site between nuclear and cytosolic mRNA libraries.
Figure S16. Similar nucleotides composition between the poly(A) sites and the control sites.
Figure S17. A similar but weaker structure feature across the alternative polyadenylation sites.
Figure S18. An accumulation of the conventional polyadenylation signal (PAS) motif "AAUAAA" in the -28 nt to -17 nt upstream of the poly(A) sites.

Table S1. Summary of reads mapping for each library.
Table S2. Comparison between SHAPE reactivity profiles and phylogenetic U1 and U12 snRNA structures.
Table S3. Summary of primer sequences.

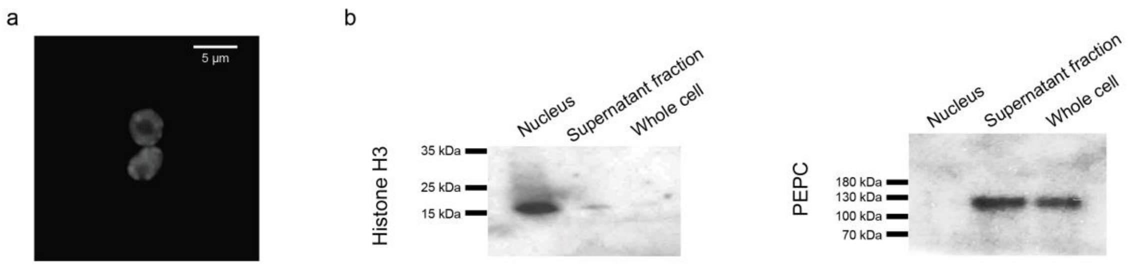

Figure S1. Assessment of nucleus isolation.
a, The microscopy image of isolated nuclei with DAPI staining is shown. The nuclei remain completely intact. **b,** The western blot of the extracted proteins from isolated nuclei, cytosolic supernatant fraction and whole cells is shown for detecting the histone H3 and cytoplasm specific protein PEPC. The high enrichment of histone H3 and the absence of PEPC indicates the purity of the isolated nucleus.

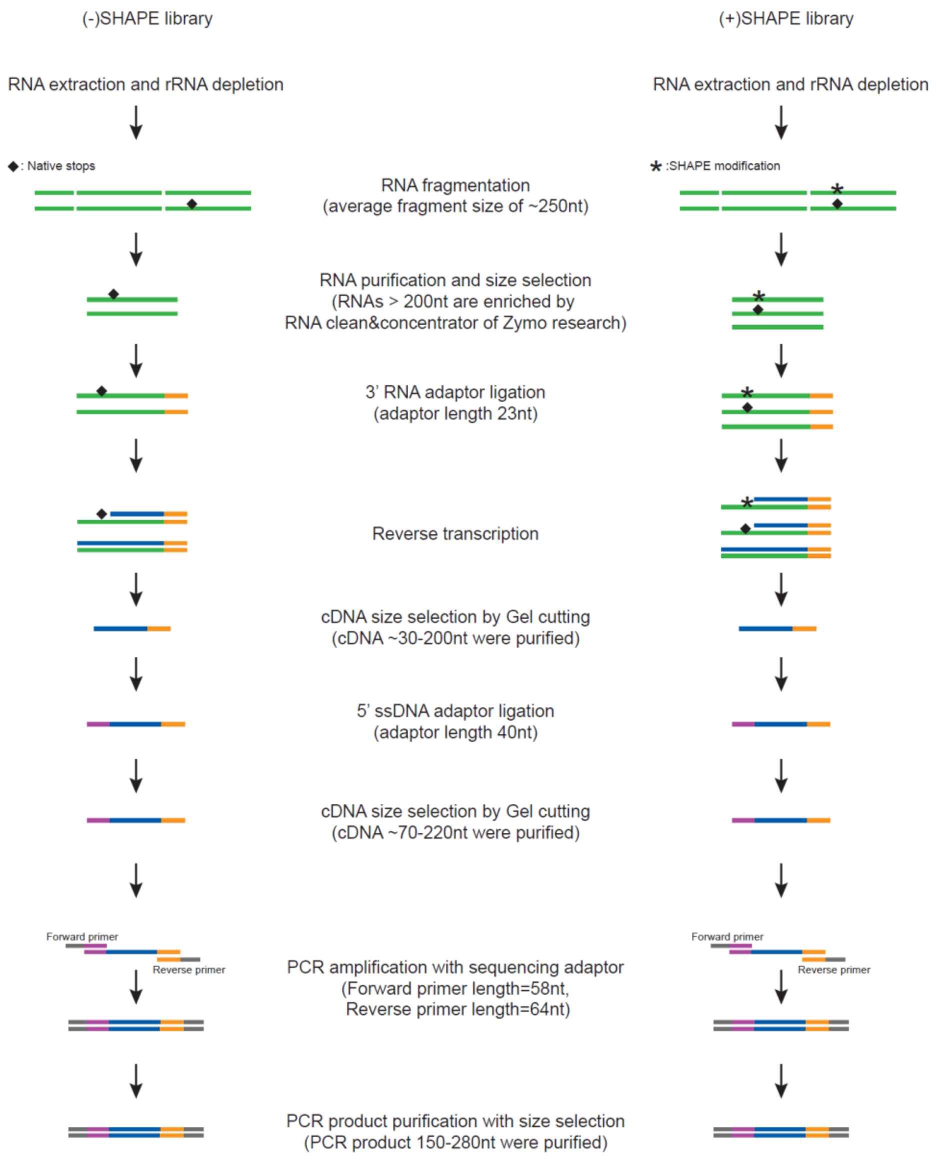

Figure S2. The step-by-step procedure of Nuc-SHAPE-Structure-Seq library construction. Both (-)SHAPE and (+)SHAPE Structure-Seq libraries were constructed using the same procedure. After RNA extraction and ribosomal RNA depletion, RNA fragmentation was performed and yielded an average fragment size of ~250nt. RNA clean and concentrator was used to purify and recover the RNA fragments with length >200nt. Following RNA purification and recovery, 3'dephosphorylation and 3′ RNA adaptor ligation was performed. Following reverse transcription, the first round of 10% TBE-Urea gel purification was performed. The size from between 30nt (longer than the size of 3'adaptor) to 200nt (~20 nt less than the smallest RNA fragment size plus the size of 3'adaptor) was selected. The size-selected cDNAs were then purified and ligated with the 5' ssDNA adaptor. After ligation, a second round of 10% TBE-Urea gel purification was performed to select the size from between 70nt (the smallest cDNA size plus the size of 5'adaptor) to 220 nt (the largest cDNA size plus the size of 5'adaptor). PCR reaction and agarose gel purification were performed. The dsDNA libraries with the size ranging from 150 nt (the smallest ligated cDNA size plus PCR primers size) to 280nt (the largest ligated cDNA size plus PCR primers size) were subjected to the deep sequencing. Black diamond represents native stop, black asterisk represents SHAPE chemical modification.

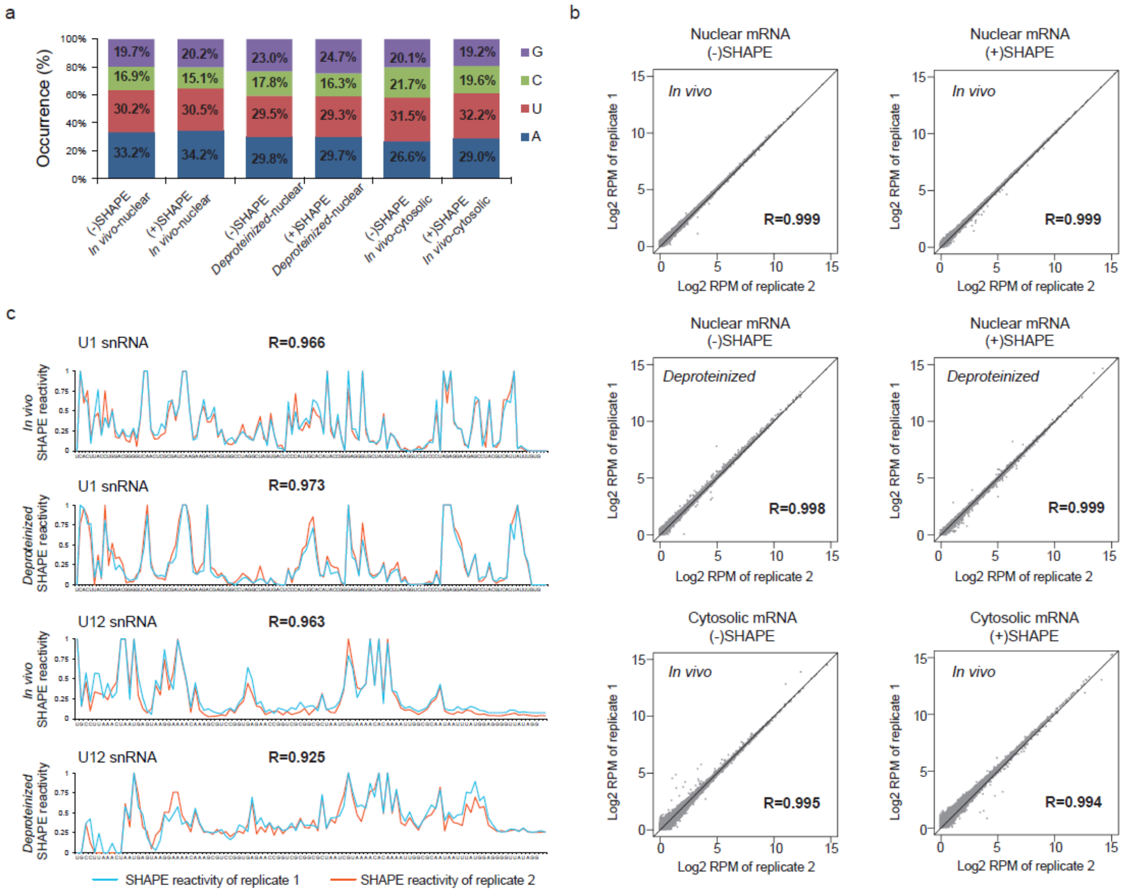

Figure S3. Summary of SHAPE-Structure-Seq libraries.

a, Nucleotide modification in both (+)SHAPE and (-)SHAPE libraries are highly concordant, with a slight enrichment in (+)SHAPE for As and Us over Cs and Gs as expected. Nucleotide occurrences are shown in both (+)SHAPE and (-)SHAPE libraries for *in vivo* nuclear SHAPE-Structure-Seq, *deproteinized* nuclear SHAPE-Structure-Seq and *in vivo* cytosolic SHAPE-Structure-Seq. **b,** Correlation of gene expression abundance between the two biological replicates for *in vivo* nuclear SHAPE-Structure-Seq, *deproteinized* nuclear SHAPE-Structure-Seq and *in vivo* cytosolic SHAPE-Structure-Seq. High correlations of gene expression abundance indicate high reproducibility between the two biological replicates. RPM: reads per million mapped reads. Pearson correlation coefficients are shown for each comparison. **c,** Correlation of SHAPE reactivity values between the two biological replicates for U1 and U12 snRNA in *in vivo* and *deproteinized* nuclear SHAPE-Structure-Seq. High SHAPE reactivity correlations indicate high reproducibility of SHAPE modification between the two biological replicates. Pearson correlation coefficients are shown for each comparison.

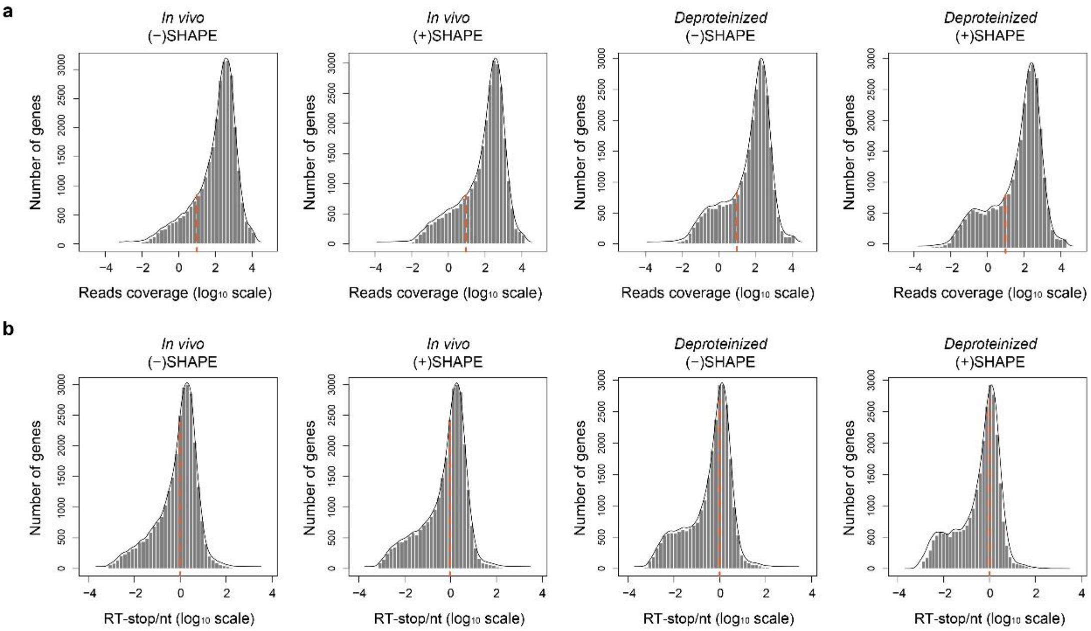

Figure S4. High reads coverage of the nuclear SHAPE-Structure-Seq libraries.

a, Over 25,051 and 22,945 genes have the sequencing reads coverage for more than 10 reads per nucleotide for (-)SHAPE and (+)SHAPE libraries in the *in vivo* nuclear SHAPE-Structure-Seq respectively. Over 20,752 and 21,141 genes have the sequencing reads coverage for more than 10 reads per nucleotide for (-)SHAPE and (+)SHAPE libraries in the *deproteinized* nuclear SHAPE-Structure-Seq respectively. **b,** Over 17,441 and 16,084 genes have more than one RT stop per nucleotide for (-)SHAPE and (+)SHAPE libraries in the *in vivo* nuclear SHAPE-Structure-Seq respectively. Over 12,366 and 13,791 genes have more than one RT stop per nucleotide for (-)SHAPE and (+)SHAPE libraries in the *deproteinized* nuclear SHAPE-Structure-Seq respectively.

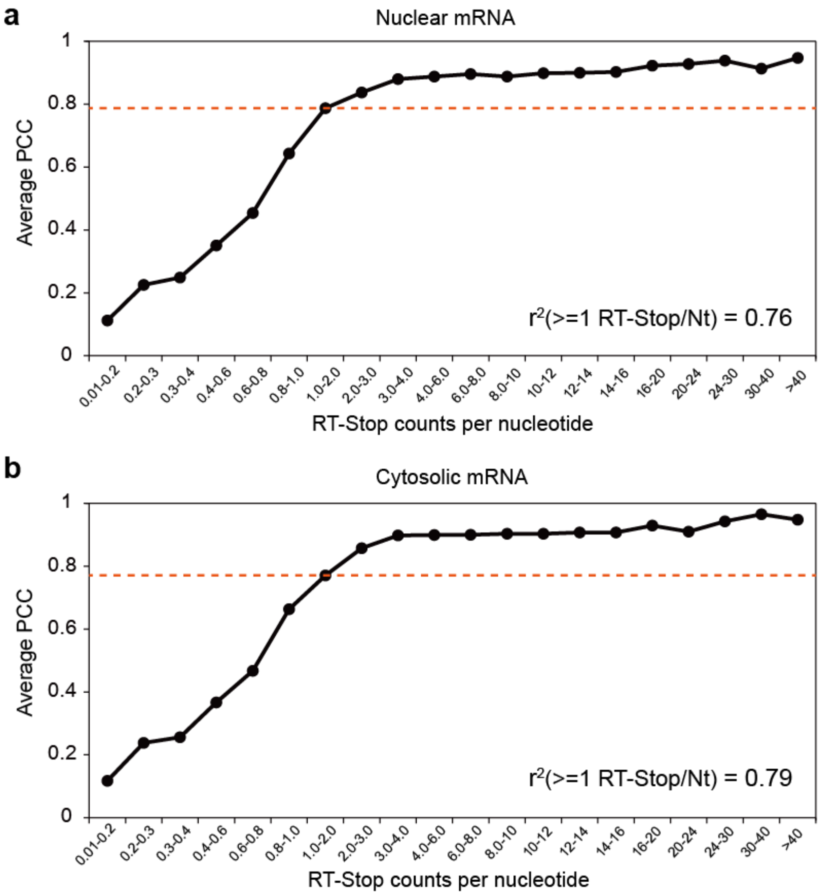

Figure S5. Average Pearson correlation coefficient (PCC) of SHAPE reactivities between the two biological replicates of the *in vivo* nuclear and cytosolic SHAPE Structure-seq libraries for mRNAs with different RT-stop reads counts.

Average Pearson correlation coefficient (PCC) values are shown for transcripts with different sequencing depth (reverse transcription stop (RT-stop) counts per nucleotide) in both *in vivo* nuclear (**a**) and cytosolic SHAPE-Structure-Seq libraries (**b**). The dash lines indicate the threshold of more than one RT-stop count per nucleotide. The r^2 of the average PCC for mRNAs with more than one RT-stop count per nucleotide was shown in each plot.

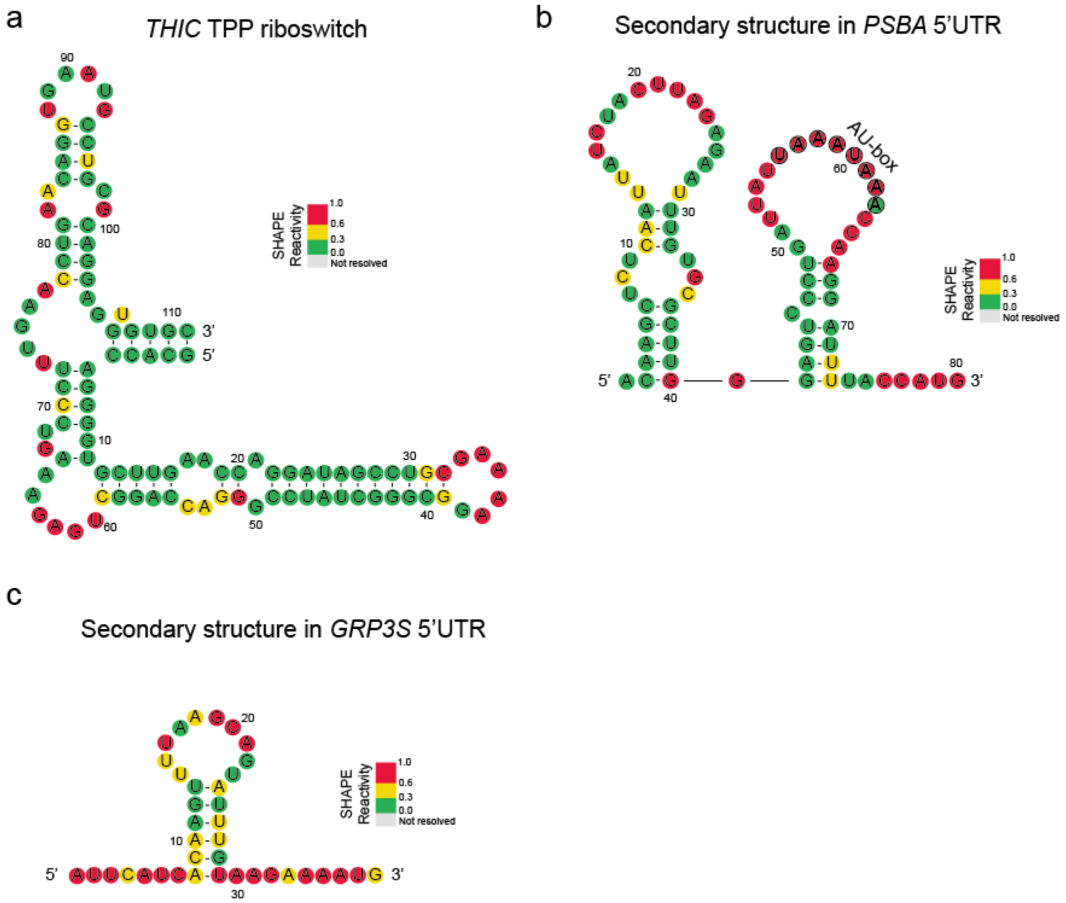

Figure S6. Comparison of SHAPE reactivity profiles with previously published mRNA secondary structure models.

a, The evolutionary conserved RNA secondary structure model of *Arabidopsis THIC* pre-mRNA (TPP (thiamin pyrophosphate) riboswitch) before splicing [1]. *In vivo* SHAPE reactivity values from our Nuc-SHAPE-Structure-Seq agree well with the structure model. Nucleotides were colour-coded according to *in vivo* SHAPE reactivity values (SHAPE reactivity 0.6-1.0 marked in red, 0.3-0.6 marked in yellow, 0-0.3 marked in green). **b,** A *in vivo* RNA secondary structure model in the 5'UTR of Arabidopsis chloroplast gene *PSBA* (*ATCG00020*). SHAPE reactivity values from our SHAPE-Structure-Seq agree with the previously-identified structure model. The AU-box (denoted by circled nucleotides) was found to be single-stranded, which is consistent with previous study [2]. **c,** A *in vivo* secondary structure model in the 5′UTR of *Arabidopsis GRP3S* (*AT2G05380*) determined by DMS-LMPCR in a previous study [2]. SHAPE reactivity values from our SHAPE-Structure-Seq agree with the structure model.

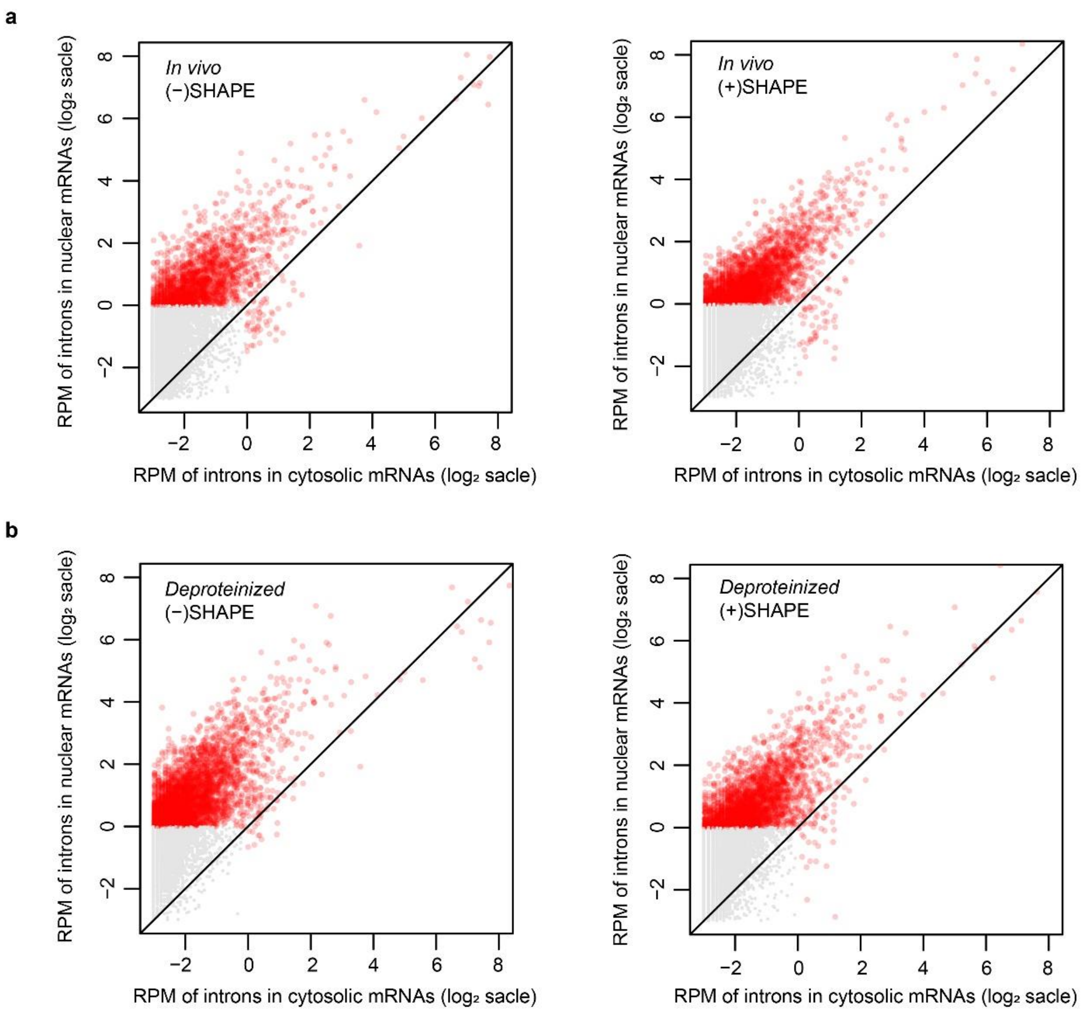

Figure S7. The high enrichment of pre-mRNAs in the nuclear SHAPE-Structure-Seq libraries.

Comparisons of the expression abundance for constitutively spliced introns between nuclear and cytosolic mRNAs in both (-)SHAPE and (+)SHAPE libraries for *in vivo* **(a)** and *deproteinized* **(b)** nuclear SHAPE-Structure-Seq. RPM: reads per million mapped reads. Introns with RPM <1 (low expression) in both nuclear mRNA and cytosolic mRNA libraries are colored in grey. Introns with RPM >=1 (high expression) in either nuclear mRNA or cytosolic mRNA libraries are colored in red. Introns are high enriched in our nuclear SHAPE-Structure-Seq libraries compared to cytosolic SHAPE-Structure-Seq libraries.

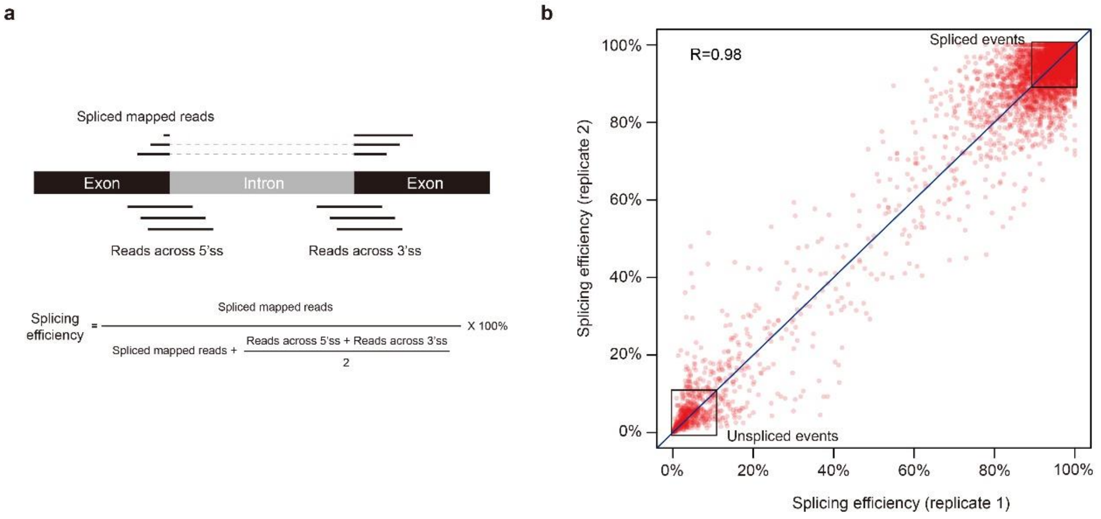

Figure S8. The formula for calculating the splicing efficiency and the identification of spliced and unspliced events.

a, The formula for calculating the splicing efficiency is shown. Higher splicing efficiency represents that the intron is more efficiently removed from pre-mRNA. **b**, The identification of spliced and unspliced events. Splicing efficiency calculated from the two biological replicates are shown. High correlation between these two replicates is observed (Pearson correlation coefficient = 0.98). Most of the introns show either very high splicing efficiency (>=90%) or very low splicing efficiency (<=10%). Two groups of splicing events were identified: spliced events (splicing efficiency >= 90%) and unspliced events (splicing efficiency <= 10%), which are highlighted in black box.

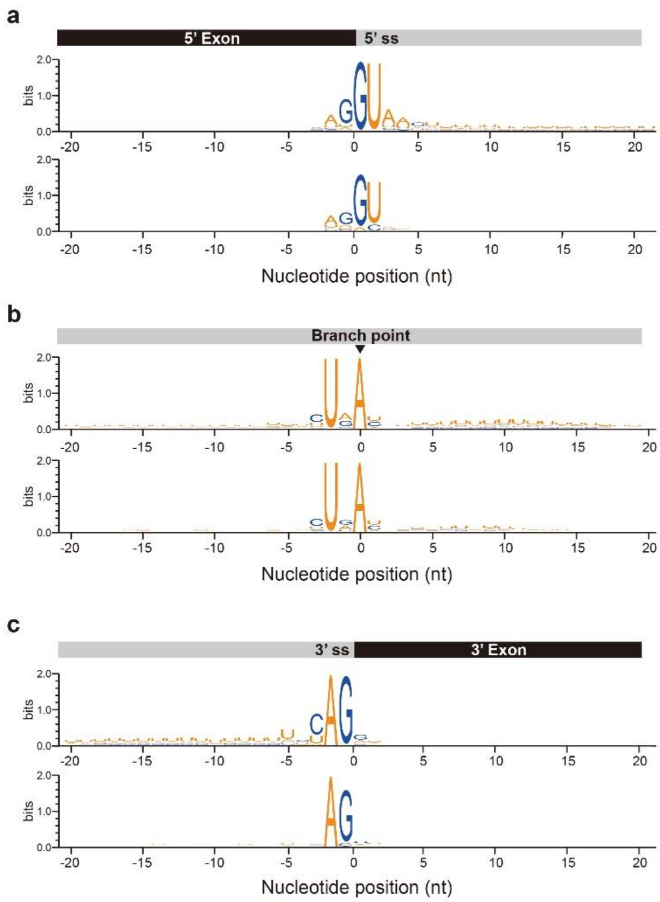

Figure S9. Similar nucleotide composition between spliced and unspliced events. Nucleotide composition across 5'ss (**a**), branch point (**b**) and 3'ss (**c**) between spliced (top) and unspliced (bottom) events are shown.

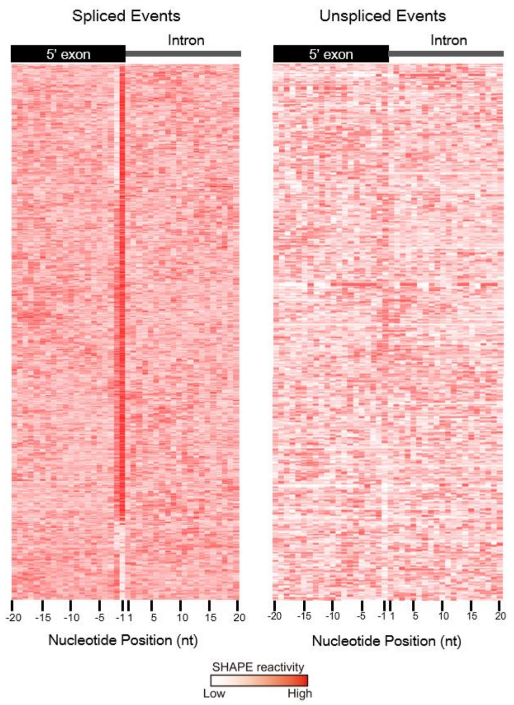

Figure S10. Heatmaps showing the SHAPE reactivities across 5'ss for spliced and unspliced events.

In vivo SHAPE reactivities across 5'ss were color coded for each individual mRNAs and shown in heatmaps. The gradient color from light to dark red represents SHAPE reactivities from low to high.

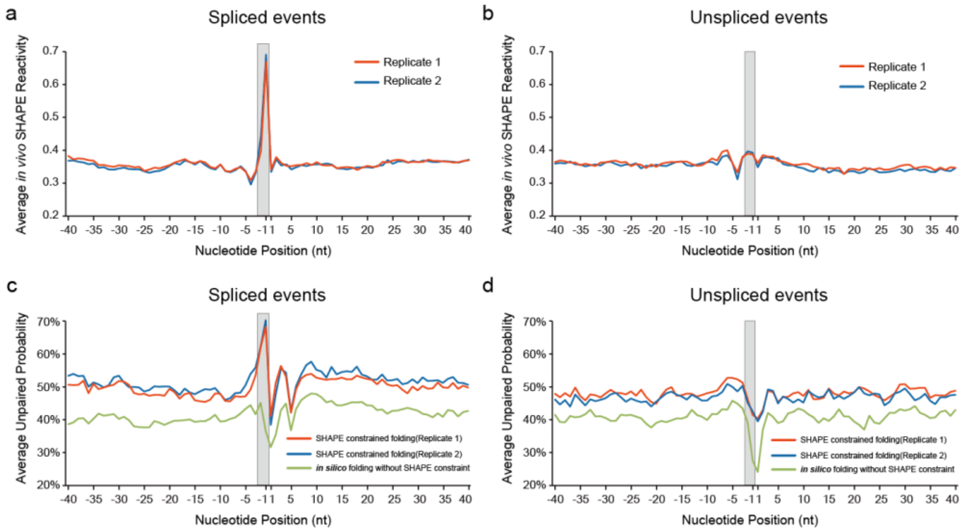

Figure S11. SHAPE reactivity and unpaired probability profiles of the two biological replicates across 5' splice site.

SHAPE reactivity profiles of the two biological replicates across 5'ss were plotted for spliced events (**a**) and unspliced events (**b**). SHAPE reactivity constrained and non-SHAPE constrained RNA structure folding was performed. Unpaired probability profiles across 5'ss derived from the SHAPE constrained RNA structures and the non-SHAPE constrained RNA structures were shown for spliced events (**c**) and unspliced events (**d**). The 100nt sequences flanking 5'ss were used in RNA structure folding by *RNAfold* in Vienna package [3]. The -1 and -2 positions upstream of 5'ss are shaded in grey.

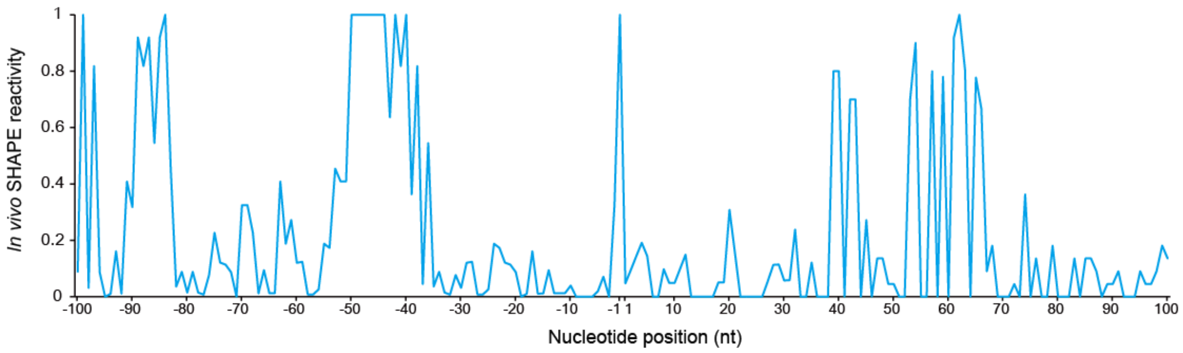

Figure S12. The SHAPE reactivity values across the 5'ss of the first intron of *AT5G56870*.
The x-axis represents the relative positions across the 5'ss.

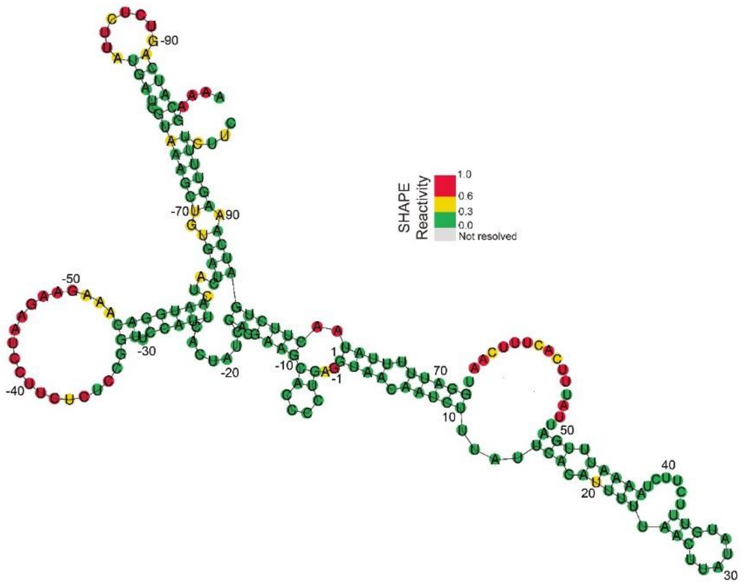

Figure S13. Minimum free energy RNA structure at the 5'ss of *AT5G56780* intron 1 folded by *RNAfold*.
The minimum free energy (MFE) RNA structure for the exon-intron region of *AT5G56780* intron 1 was generated by *RNAfold* program in *ViennaRNA* Package. The *in vivo* SHAPE reactivity values are color-coded and aligned to the MFE RNA structure (SHAPE reactivity 0.6-1.0 marked in red, 0.3-0.6 marked in yellow, 0-0.3 marked in green). The -1 and -2 positions are all single-stranded in the MFE RNA structure.

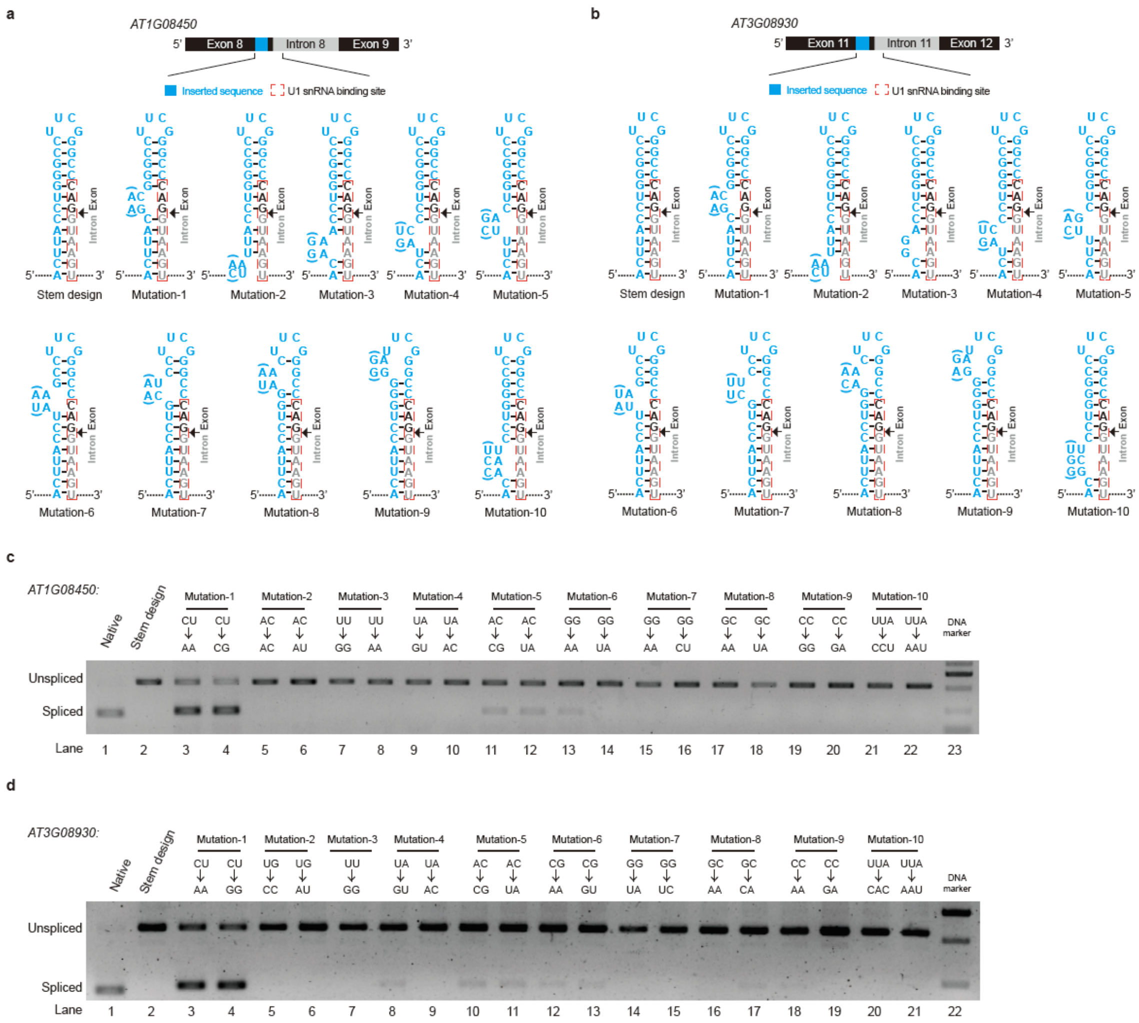

Figure S14. The two-nucleotide single-stranded RNA structure feature at -1 and -2 nt positions upstream of 5'ss can regulate splicing on the genes *AT1G08450* and *AT3G08930*
a,b, Schematic of experimental design to validate the effect of single-strandedness at the -1 and -2 positions of 5'ss on splicing of *AT1G08450* (**a**) and *AT3G08930* (**b**). A short sequence (blue) was inserted immediately upstream of the U1 snRNA binding site (red dashed box) to form a stable hairpin structure with the whole U1 binding site completely base-paired. The exon and intron sequences are colored in black and grey, respectively. A series of mutations were introduced at different positions of the inserted sequence to disrupt the base-pairing status of different nucleotides. Two types of mutations (with/without brackets) were designed for each position to avoid potential effects due to changing the sequence content. We introduced only one type of mutation for mutation-3 "GG" in *AT3G08930,* because the corresponding thermodynamic structure prediction shows that other types of mutations will lead to alternative long-distance interactions with flanking sequence content. **c,d,** Determination of splicing events by transient expression assay in *Nicotiana benthamiana*. The spliced and unspliced products were distinguished by semiquantitative RT-PCR using the same pair of primers located upstream and downstream of the intron. Spliced and unspliced products are indicated by bands with different sizes. The splicing events of *AT1G08450* (**c**) and *AT3G08930* (**d**) are shown. The constructs with native sequences were successfully spliced (lane 1). The splicing was completely inhibited in the stem design (lane 2). The mutations disrupted the base-pairing status at -1 and -2 positions upstream of 5'ss (Mutation-1) and rescued the splicing (lane 3 and 4). All other mutations (Mutation 2-10) did not rescue the splicing (lanes 5-22).

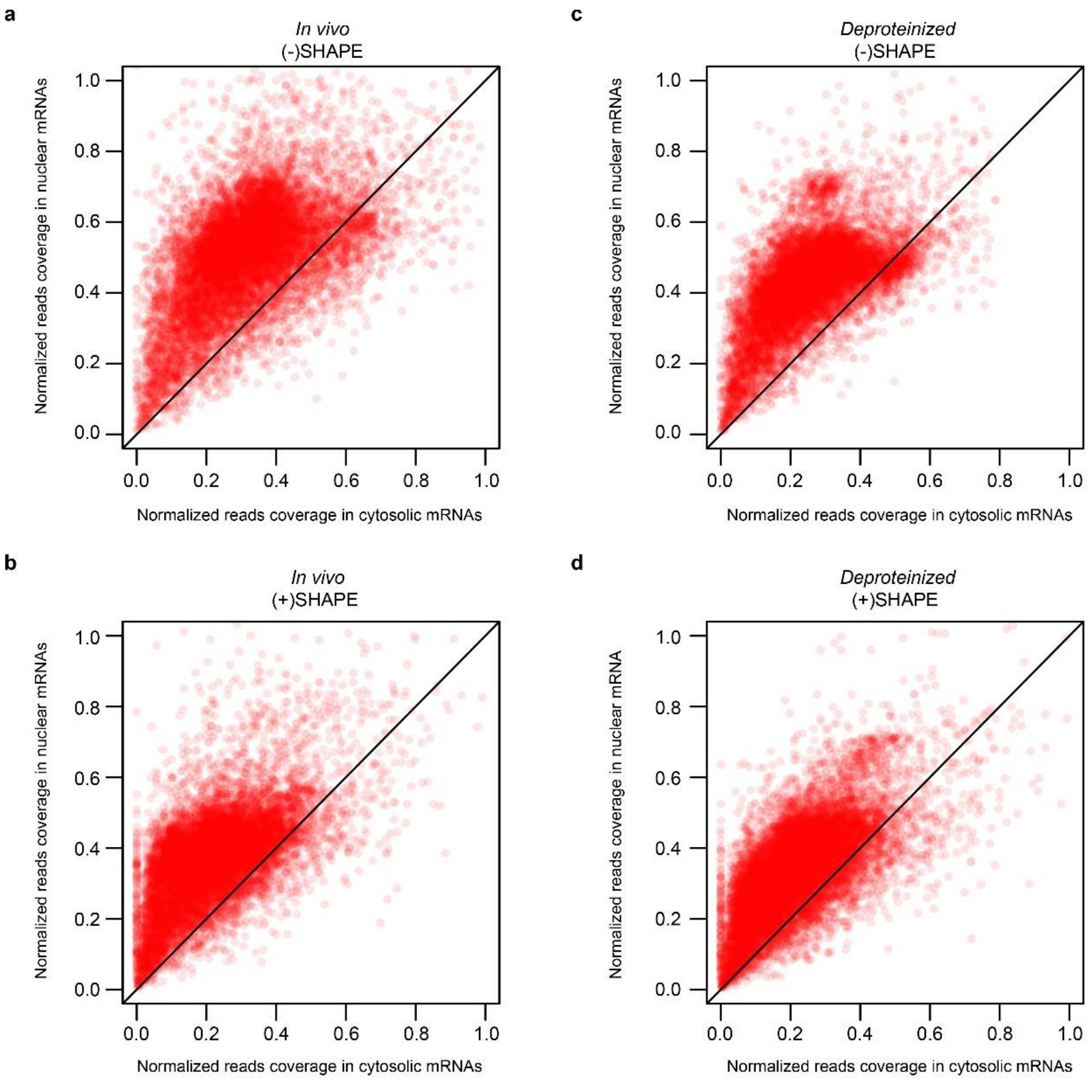

Figure S15. Comparison of reads coverage across poly(A) site between nuclear and cytosolic mRNA libraries.

To compare poly(A) site reads coverage between different libraries, reads coverage across the poly(A) site of each gene was normalized to the average reads coverage of the corresponding gene. Comparisons of normalized reads coverage between nuclear and cytosolic mRNAs in both (-)SHAPE and (+)SHAPE libraries for *in vivo* **(a, b)** and *deproteinized* **(c, d)** conditions. Higher reads coverage across the poly(A) sites in nuclear SHAPE-Structure-Seq libraries compared to cytosolic SHAPE-Structure-Seq libraries indicates the high enrichment of pre-mRNA before polyadenylation in nuclear mRNAs.

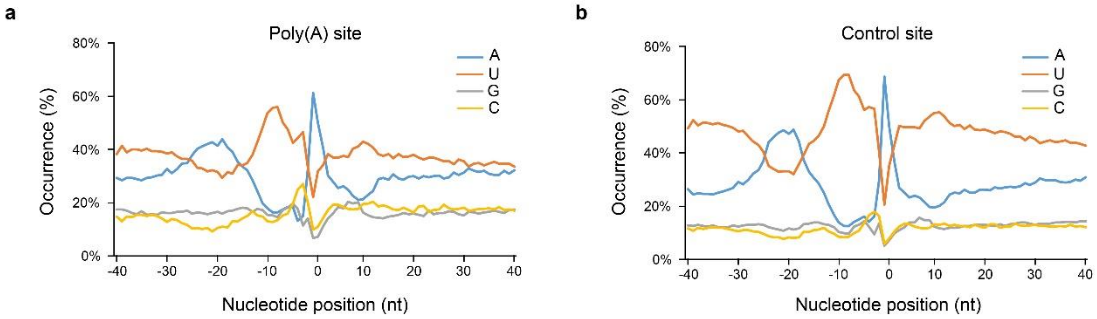

Figure S16. Similar nucleotides composition between the poly(A) sites and the control sites. **a,** Nucleotide composition profile across the poly(A) sites. **b,** Nucleotide composition profile across the control sites.

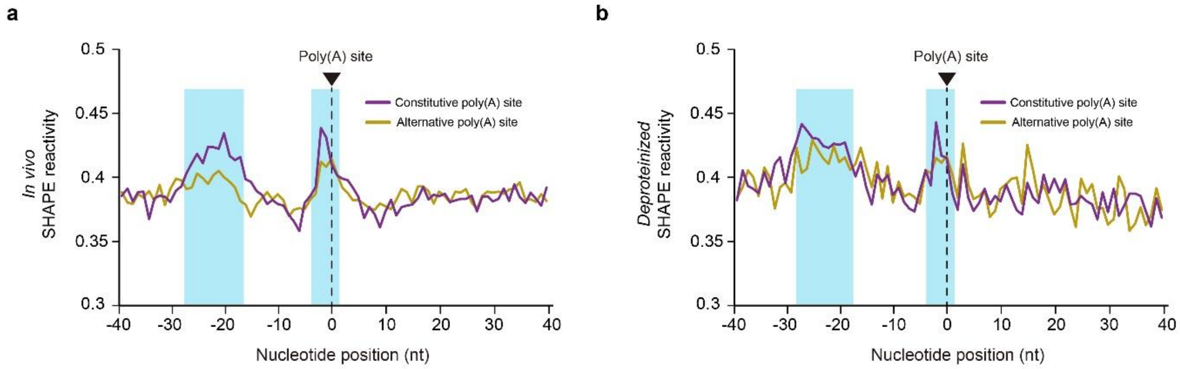

Figure S17. A similar but weaker structure feature across the alternative polyadenylation sites.

a, b, Average SHAPE reactivity profiles across both the constitutive poly(A) sites (purple) and alternative poly(A) (stone yellow) sites for *in vivo* (**a**) and *deproteinized* (**b**) conditions are shown. The X-axis represents the relative position to the poly(A) site. The average SHAPE reactivities in two regions (from -28 nt to -17 nt upstream of the poly(A) site and from -4 nt to +1 nt across the poly(A) site, shaded in light blue) across the constitutive poly(A) (purple) sites are similar with those across the alternative poly(A) (stone yellow) sites.

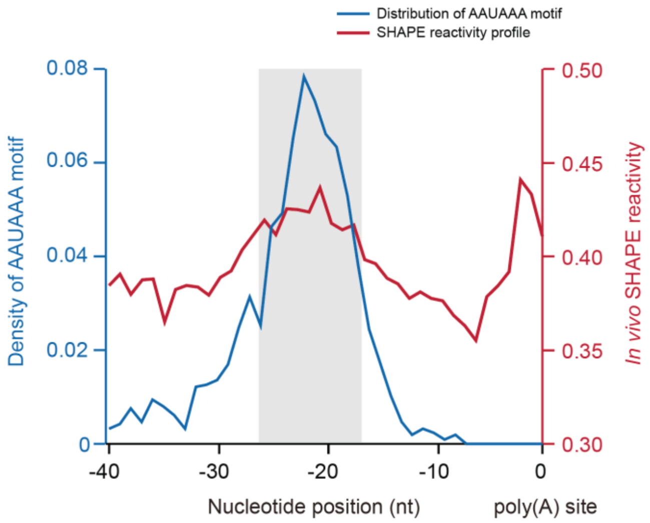

Figure S18. An accumulation of the conventional polyadenylation signal (PAS) motif "AAUAAA" in the -28 nt to -17 nt upstream of the poly(A) sites.

The "AAUAAA" motif distribution upstream of poly (A) sites is plotted as the blue line while *in vivo* SHAPE reactivity profile is plotted as the red line. An accumulation of the conventional polyadenylation signal (PAS) motif "AAUAAA" is in the -28 nt to -17 nt (shaded in light grey) upstream of the poly(A) sites.

Table S1. Summary of reads mapping for each library.

Libraries	Biological replicates	Sequencing depth	No. of mapped reads	% of mapped reads
Nuclear RNA (*deproteinized*) (+)SHAPE	1	339,824,973	233,350,092	69%
Nuclear RNA (*deproteinized*) (+)SHAPE	2	361,592,757	244,657,771	68%
Nuclear RNA (*deproteinized*) (-)SHAPE	1	169,654,866	147,979,243	87%
Nuclear RNA (*deproteinized*) (-)SHAPE	2	167,190,546	146,873,727	88%
Nuclear RNA (*in vivo*) (+)SHAPE	1	341,438,706	283,762,996	83%
Nuclear RNA (*in vivo*) (+)SHAPE	2	296,287,026	247,523,784	84%
Nuclear RNA (*in vivo*) (-)SHAPE	1	447,447,740	372,396,093	83%
Nuclear RNA (*in vivo*) (-)SHAPE	2	342,500,235	294,505,102	86%
Cytosolic RNA (*in vivo*) (+)SHAPE	1	46,421,231	31,691,004	68%
Cytosolic RNA (*in vivo*) (+)SHAPE	2	58,082,808	38,195,080	66%
Cytosolic RNA (*in vivo*) (-)SHAPE	1	61,192,363	45,426,877	74%
Cytosolic RNA (*in vivo*) (-)SHAPE	2	59,907,555	40,821,132	68%

Table S2. Comparison between SHAPE reactivity profiles and phylogenetic U1 and U12 snRNA structures. Ture positive (proportion of single-stranded nucleotides in high SHAPE reactive nucleotides), true negative (proportion of double-stranded nucleotides in low SHAPE reactive nucleotides), false positive (proportion of double-stranded nucleotides in high SHAPE reactive nucleotides) and false negative (proportion of single-stranded nucleotides in low SHAPE reactive nucleotides) values were calculated and shown. Some reactive nucleotides that are annotated as base-paired in the phylogenetic structure but positioned either at the end of a helix or adjacent to a helical defect such as a bulge or loop, locations that are known to lead to flexibility. Values in parentheses, corrected for this positioning, show higher true positive and lower false positive percentages.

		High SHAPE reactivity (>0.6)	Low SHAPE reactivity (<0.3)
U1 snRNA (*in vivo*)	Single-stranded	88% (96.0%)	17.1%
	Double-stranded	12% (4.0%)	82.9%
U12 snRNA (*in vivo*)	Single-stranded	84.6% (92.3)	26.2%
	Double-stranded	15.4% (7.7%)	73.8%
U1 snRNA (*deproteinized*)	Single-stranded	90.9% (95.5%)	26.9%
	Double-stranded	9.1% (4.5%)	73.1%
U12 snRNA (*deproteinized*)	Single-stranded	88.5% (92.3%)	19.6%
	Double-stranded	11.5% (7.7%)	80.4%

Table S3. Summary of primer sequences

Red underlined text indicates reverse index sequence. Blue underlined text indicates the mutated nucleotides.

Primer name	Sequence (5'-3')
DNA for 3'adapter ligation	/5rApp/AGATCGGAAGAGCACACGTCTG/3SpC3/
DNA for library reverse transcription	CAGACGTGTGCTCTTCCGATCT
DNA for 5'adapter ligation	/5Phos/AGATCGGAAGAGCGTCGTGTAGCTCTTCCGATCTNNNNNN/3SpC3/
Forward primer for sequencing Library	AATGATACGGCGACCACCGAGATCTACACTCTTTCCCTACACGACGCTCTTCCGATCT
Reverse primer for sequencing (Index2) Library	CAAGCAGAAGACGGCATACGAGATACATCGGTGACTGGAGTTCAGACGTGTGCTCTTCCGATCT
Reverse primer for sequencing (Index3) Library	CAAGCAGAAGACGGCATACGAGATGCCTAAGTGACTGGAGTTCAGACGTGTGCTCTTCCGATCT
Reverse primer for sequencing (Index4) Library	CAAGCAGAAGACGGCATACGAGATTGGTCAGTGACTGGAGTTCAGACGTGTGCTCTTCCGATCT
Reverse primer for sequencing (Index5) Library	CAAGCAGAAGACGGCATACGAGATCACTGTGTGACTGGAGTTCAGACGTGTGCTCTTCCGATCT
Reverse primer for sequencing (Index6) Library	CAAGCAGAAGACGGCATACGAGATATTGGCGTGACTGGAGTTCAGACGTGTGCTCTTCCGATCT
Reverse primer for sequencing (Index7) Library	CAAGCAGAAGACGGCATACGAGATGATCTGGTGACTGGAGTTCAGACGTGTGCTCTTCCGATCT
Reverse primer for sequencing (Index21) Library	CAAGCAGAAGACGGCATACGAGATCGAAACGTGACTGGAGTTCAGACGTGTGCTCTTCCGATCT
Reverse primer for sequencing (Index25) Library	CAAGCAGAAGACGGCATACGAGATATCAGTGTGACTGGAGTTCAGACGTGTGCTCTTCCGATCT
Forward primer *AT5G56870* Minigene	AATCTGAGTTTTTCTGATTAACAGATGGTGATCATCAATGGAC
Reverse primer *AT5G56870* Minigene	TTTTGGCGTCTTCCATCCCTCATTGTCCAGGAGAAGGT
Forward primer *AT1G0845* Minigene	AATCTGAGTTTTTCTGATTAACAGATGTTTGAAGATGATCCAGA
Reverse primer *AT1G0845* Minigene	TTTTGGCGTCTTCCATCCCTTACTCCCTGTGTTGGGCA
Forward primer *AT3G08930* Minigene	AATCTGAGTTTTTCTGATTAACAGATGTGGGGAGCTACTCTAAT
Reverse primer *AT3G08930* Minigene	TTTTGGCGTCTTCCATCCCTTGTAAAGGTACTTAATTCC
Forward primer *AT5G56870*_Semi-qPCR	AATCTGAGTTTTTCTGATTAACAGATGGTGATCATCAATGGAC
Reverse primer *AT5G56870*_Semi-qPCR	TTTTGGCGTCTTCCATCCCTCATTGTCCAGGAGAAGGT
Forward primer *AT1G0845*_Semi-qPCR	ATGTTTGAAGATGATCCAGA
Reverse primer *AT1G0845*_Semi-qPCR	TTACTCCCTGTGTTGGGCA
Forward primer *AT3G08930*_Semi-qPCR	ATGTGGGGAGCTACTCTAAT
Reverse primer *AT3G08930*_Semi-qPCR	TTGTAAAGGTACTTAATTCC
Forward primer *AT5G56870*_Mut1 CU-AA	CCCTTGTTACAACGGCCTTCGG
Reverse primer *AT5G56870*_Mut1 CU-AA	GTGCTTCGTGGATAGTGAATG
Forward primer *AT5G56870*_Mut1 CU-GG	CCCTTGTTACGGCGGCCTTCGG
Reverse primer *AT5G56870*_Mut1 CU-GG	GTGCTTCGTGGATAGTGAATG
Forward primer *AT5G56870*_Mut2 UA-GU	CACCCCTTGTGTCCTCGGCCTT
Reverse primer *AT5G56870*_Mut2 UA-GU	CTTCGTGGATAGTGAATGG
Forward primer *AT5G56870*_Mut2 UA-AC	CACCCCTTGTACCCTCGGCCTT
Reverse primer *AT5G56870*_Mut2 UA-AC	CTTCGTGGATAGTGAATGG
Forward primer *AT5G56870*_Mut3 UU-GG	GCACCCCTTGGGACCTCGGCCT
Reverse primer *AT5G56870*_Mut3 UU-GG	TTCGTGGATAGTGAATGGAACC
Forward primer *AT5G56870*_Mut3 UU-AC	GCACCCCTTGACACCTCGGCCT
Reverse primer *AT5G56870*_Mut3 UU-AC	TTCGTGGATAGTGAATGGAACCG
Forward primer *AT5G56870*_Mut4 UG-CC	AAGCACCCCTCCTTACCTCGGC
Reverse primer *AT5G56870*_Mut4 UG-CC	CGTGGATAGTGAATGGAAC
Forward primer *AT5G56870*_Mut4 UG-AU	AAGCACCCCTATTTACCTCGGC
Reverse primer *AT5G56870*_Mut4 UG-AU	CGTGGATAGTGAATGGAAC
Forward primer *AT5G56870*_Mut5 UUA-CAC	GCACCCCTTGCACCCTCGGCCTT
Reverse primer *AT5G56870*_Mut5 UUA-CAC	TTCGTGGATAGTGAATGGAAC
Forward primer *AT5G56870*_Mut5 UUA-AAU	GCACCCCTTGAAUCCTCGGCCTT
Reverse primer *AT5G56870*_Mut5 UUA-AAU	TTCGTGGATAGTGAATGGAAC

References

1. Wachter A, Tunc-Ozdemir M, Grove BC, Green PJ, Shintani DK, Breaker RR. Riboswitch control of gene expression in plants by splicing and alternative 3' end processing of mRNAs. Plant Cell. 2007;19(11):3437-3450.
2. Kwok CK, Ding Y, Tang Y, Assmann SM, Bevilacqua PC. Determination of in vivo RNA structure in low-abundance transcripts. Nat Commun. 2013;4(2971).
3. Lorenz R, Bernhart SH, Honer Zu Siederdissen C, Tafer H, Flamm C, Stadler PF, Hofacker IL. ViennaRNA Package 2.0. Algorithms Mol Biol. 2011;6(26).
