# Supplementary material for: In vivo nuclear RNA structurome reveals RNA-structure regulation of mRNA processing in plants
Source: Genome Biol. 2021 Jan 4;22:11. doi: 10.1186/s13059-020-02236-4 (PMC7784297; doi:10.1186/s13059-020-02236-4)
Supplement: Supplementary file 10 — Additional file 10. Review history. [file 13059_2020_2236_MOESM10_ESM.docx]

Review History

**First round of review**

**Reviewer 1**

**Are you able to assess all statistics in the manuscript, including the appropriateness of statistical tests used?**

No, I do not feel adequately qualified to assess the statistics.

**Comments to author:**

In this manuscript, Ding and co-workers describe a new approach to genome-wide RNA structure probing. The authors perform in vivo Selective 2'-Hydroxyl Acylation analysed by Primer Extension (SHAPE) analysis on Arabidopsis seedlings using 2-methylnicotinic acid imidazolide (NAI), a chemical that allows probing of all nucleotides. This contrasts with the more widely used dimethyl sulfate (DMS) method, which only provides structure information of As and Cs.

The study compares the RNA structurome of nuclear and cytoplasmic cellular fractions, revealing marked differences that suggest important roles for RNA secondary structures in key biological processes. For example, exons are found to be more accessible in RNAs in the cytoplasm, thereby presumably allowing access to the translation machinery. Moreover, single-stranded RNA structures at 5' splice sites and branch sites can be important for mRNA splicing and alternative splicing, as demonstrated with transient assays in tobacco leaves using an exon-intron junction of a particular gene. Structure elements surrounding polyadenylation signal motifs and polyadenylation sites are also described.

The manuscript is well written and reports a solid body of data that provides important new insight into how RNA structure influences basic molecular processes in the plant cell. The most striking concern is that the global functional significance of structure elements at the 5' splice site in mRNA splicing is supported solely by a single example. This and other suggested improvements are listed below.

1. To strengthen the claim of a general role in splicing for single strandedness at the 5' splice site, functional validation of additional intron-exon splicing events should be presented.

2. Lines 816-818: The SHAPE treatment description in the legend of Figure 1 is confusing. It should be made more clear that deproteinized samples are only treated with NAI after nuclei isolation.

Minor comments:

3. In panel a of Figure S3, nucleotide modification differences are hard to follow. This could be improved by including the percentage values within the bars.

4. Lines 88 and 89: provide the full names of nucleotides for the abbreviations As, Cs etc.

5. Typo in Line 198: "reactivits"

6. Lines 569 and 891: "semi-qPCR" should be "semiquantitative RT-PCR".

**Reviewer 2**

**Are you able to assess all statistics in the manuscript, including the appropriateness of statistical tests used?**

No, I do not feel adequately qualified to assess the statistics.

**Comments to author:**

Liu et.al use chemical probing (SHAPE) to probe the genome-wide nuclear and cytoplasmic structures of Arabidopsis with and without proteinase treatment.

Their main claims are

1. Nuc-SHAPE generates in vivo RNA structure landscape with high accuracy.

2. Nuclear structures are globally different than cytosolic structures

3. 2-nt single stranded RNA is present upstream at splice sites (ss) and sufficient to regulate splicing

4. The polyadenylation signal (PAS) and poly(A) sites are generally open and that promotes recognition.

These claims are of broad interest but I find that the data is weak, the necessary controls are not included, and the conclusions are not adequately supported. Here are main specific concerns for each of the above four claims.

(1). There isn't convincing evidence that the RNA structure models are highly accurate or reproducible. The authors show good data for very abundant, extremely structured RNAs (U1, U12). There are no positive control mRNA structures and positive control structures in low abundant messages.

The authors need to provide justification for using the coverage cut off they did.

Some essential comparisons and data quality metrics are missing:

-	The Pearson correlation of the SHAPE signal for replicates across each message (or a given window of nucleotides) as a function of the massage abundance; that would be one way to choose an appropriate threshold for the minimum coverage.

-	The Pearson correlation of the SHAPE signal per message (or window) plotted for in vivo vs untreated, biological replicates, and nuclear vs cytoplasmic RNAs.

(2). This claim has very little support. The authors are averaging thousands of messages and find that one condition (nuclear vs cytoplasmic) gives them something on the order of 0.01-0.05 signal difference in average SHAPE reactivity (Fig2). That could easily be an artifact (e.g. RNA binding proteins that they didn't manage to get rid of during the proteinase treatment or simply a reproducibility artifact). For comparison, in Fig 1., the authors bin all reactivates that fall between 0.0 and 0.3 as a single color (green) to suggest that bases with signal between 0.0 and 0.3 are equivalent. However, in Fig2 the authors claim that 0.05 differences in signal is important and enough for a major result of their paper (i.e nuclear structure is different than cytoplasmic). The argument is not convincing. Further, it is very strange that the coding sequence close to the 5'UTR behaves differently than the coding sequence close to the 3'UTR, this doesn't make biological sense.

-	Fig 4a shows the reactivity of 70nt region in AT5G56870. There appears to be only 1 or 2 bases reactive and these bases are hyper reactive. This doesn't look like a structure profile; it looks like a profile of two hyper reactive bases. Are the authors suggesting that all the rest 68nt are paired? Where is the structure model for the wt gene in a window of 100-200nt ? Are these structures supported by other metrics such as evolutionary conservation or thermodynamic stability (e.g. ScanFold) ?

-	Fig 4b is interesting but a big conclusion is made based on too few experiments. For example, the authors didn't mutate any bases upstream of the GAG. This experiment requires a high throughput mutagenesis and testing multiple messages in order to conclude that the two nucleotides upstream of the ss are most important for regulating splicing, which seems unlikely to be generally true. The contribution of the surrounding sequence and structure must be rigorously tested by mutagenesis and splicing assay of multiple messages.

-	Fig.S7 - the authors clam there is no sequence difference of the splicing motifs but that is a subjective claim. The authors make a big deal out of differences in SHAPE signal of 0.05 but there is an equivalent amount of sequence variation, for example in S7a, the bottom (unspliced) events have an enrichment for AC that is not present in the top GU (spliced) sites.

(4) It is difficult to make conclusions out of these results. For example, the authors show in Fig. 5g that indeed 20/26% of the PAS site and PolyA sites are in stems. Are these sites less functional or are these sites equally good motifs as the ones that are in loops? Also fig.5a, why do the control sites have lower SHAPE reactivity baseline than any of the PolyA sites? The control sites control for sequence, they should have the same baseline, unless there is protein contamination or something else not structure (perhaps not enough sites were used in each bin to accurately reflect the average behavior), which is creating a higher SHAPE baseline for the polyA sites. The presence of multiple potential artifacts make it difficult, or impossible, to interpret the "bump" in SHAPE reactivity.

**Authors Response**

**Point-by-point responses to the reviewers’ comments:**

Reviewer #1:

In this manuscript, Ding and co-workers describe a new approach to genome-wide RNA structure probing. The authors perform in vivo Selective 2'-Hydroxyl Acylation analysed by Primer Extension (SHAPE) analysis on Arabidopsis seedlings using 2-methylnicotinic acid imidazolide (NAI), a chemical that allows probing of all nucleotides. This contrasts with the more widely used dimethyl sulfate (DMS) method, which only provides structure information of As and Cs.

The study compares the RNA structurome of nuclear and cytoplasmic cellular fractions, revealing marked differences that suggest important roles for RNA secondary structures in key biological processes. For example, exons are found to be more accessible in RNAs in the cytoplasm, thereby presumably allowing access to the translation machinery. Moreover, single-stranded RNA structures at 5' splice sites and branch sites can be important for mRNA splicing and alternative splicing, as demonstrated with transient assays in tobacco leaves using an exon-intron junction of a particular gene. Structure elements surrounding polyadenylation signal motifs and polyadenylation sites are also described.

The manuscript is well written and reports a solid body of data that provides important new insight into how RNA structure influences basic molecular processes in the plant cell. The most striking concern is that the global functional significance of structure elements at the 5' splice site in mRNA splicing is supported solely by a single example. This and other suggested improvements are listed below.

*Response:*

*We thank the Referee for their appreciation that: “The manuscript is well written and reports a solid body of data that provides important new insight into how RNA structure influences basic molecular processes in the plant cell”. We welcome their constructive suggestions to help us improve our manuscript. We have made all the suggested improvements as follows:*

1.	To strengthen the claim of a general role in splicing for single strandedness at the 5' splice site,

functional validation of additional intron-exon splicing events should be presented.

*Response:*

*We thank the referee for this suggestion. We have performed the same functional validation on two additional genes AT1G08450 and AT3G08930 (Rebuttal Figure 1 (New Figure S10)). Moreover, as suggested by reviewer #2, we have also designed more mutations upstream the 5’ splice site (5’ss) to strengthen our conclusions (Rebuttal Figure 1 (New Figure S10), Rebuttal Figure 2b,d (revised Figure 4b,d)). For all these three genes (AT5G56780, AT1G08450 and AT3G08930), only the mutations which led to the single-strandedness at -1 and -2 positions of 5’ss can significantly restore the splicing events (Rebuttal Figure 1 (New Figure S10), Rebuttal Figure 2b,d (revised Figure 4b,d)). Hence, our results confirmed that the two-nucleotide single-stranded RNA structure feature at -1 and -2 positions upstream of 5’ss is sufficient to regulate splicing.*

*Rebuttal Figure 1 (new Figure S10). The two-nucleotide single-stranded RNA structure feature at -1 and -2 nt positions upstream of 5’ss can regulate splicing on the genes AT1G08450 and AT3G08930*

*a,b, Schematic of experimental design to validate the effect of single-strandedness at the -1 and -2 positions of 5’ss on splicing of AT1G08450 (a) and AT3G08930 (b). A short sequence (blue) was inserted immediately upstream of the U1 snRNA binding site (red dashed box) to form a stable hairpin structure with the whole U1 binding site completely base-paired. The exon and intron sequences are colored in black and grey, respectively. A series of mutations were introduced at different positions of the inserted sequence to disrupt the base-pairing status of different nucleotides. Two types of mutations (with/without brackets) were designed for each position to avoid potential effects due to changing the sequence content. We introduced only one type of mutation for mutation-3 “GG” in AT3G08930, because the corresponding thermodynamic structure prediction shows that other types of mutations will lead to alternative long-distance interactions with flanking sequence content. c,d, Determination of splicing events by transient expression assay in Nicotiana benthamiana. The spliced and unspliced products were distinguished by semiquantitative RT-PCR using the same pair of primers located upstream and downstream of the intron. Spliced and unspliced products are indicated by bands with different sizes. The splicing events of AT1G08450 (c) and AT3G08930 (d) are shown. The constructs with native sequences were successfully spliced (lane 1). The splicing was completely inhibited in the stem design (lane 2). The mutations disrupted the base-pairing status at -1 and -2 positions upstream of 5’ss (Mutation-1) and rescued the splicing (lane 3 and 4). All other mutations (Mutation 2-10) did not rescue the splicing (lanes 5-22).*

*Rebuttal Figure 2 (Revised Figure 4, b,d panels have been revised according to our additional experimental design; a,c panels are the same with the original figure). The two-nucleotide single-stranded RNA structure feature at -1 and -2 nt positions upstream of 5’ss can regulate splicing. a, SHAPE reactivity profiles across 5’ss of the first intron of AT5G56870. High SHAPE reactivities are observed at -1 and -2 nt positions (shaded in yellow) upstream of 5’ss under both in vivo (top) and deproteinized (bottom) conditions, which resemble the global SHAPE reactivity profiles for spliced events. b, Schematic of experimental design to validate the effect of single-strandedness at the -1 and - 2 positions of 5’ss on splicing of the first intron of AT5G56870. A short sequence (blue) was inserted immediately upstream of the U1 snRNA binding site (red dashed box) to form a stable hairpin structure with the whole U1 binding site completely base-paired. The exon and intron sequences are colored in black and grey respectively. A series of mutations were introduced at different positions of the inserted sequence to disrupt the base-pairing status of different nucleotides. Two types of mutations (with/without bracket) were designed for each position to avoid potential effects due to changing the sequence content. c,d Determination of splicing events by transient expression assay in Nicotiana benthamiana. The spliced and unspliced products were distinguished by semiquantitative RT-PCR using the same pair of primers located upstream and downstream of the intron. Spliced and unspliced products are indicated by bands with different sizes. The construct with native sequence was successfully spliced (lane 1). The splicing was completely inhibited in the stem design (lane 2). The mutation “AA” or “GG” disrupted the base-pairing status at -1 and -2 positions upstream of 5’ss (Mutation-1) and rescued the splicing (lane 3 and 4). All other mutations (Mutation 2-10) designed to disrupt other base-pairing sites across the U1 binding site did not rescue the splicing (lanes 5-22). Lane 23, the DNA marker.*

2. Lines 816-818: The SHAPE treatment description in the legend of Figure 1 is confusing. It should be made more clear that deproteinized samples are only treated with NAI after nuclei isolation.

*Response:*

*We thank the referee for this suggestion. We have revised the manuscript on line 850-851 to:*

*“SHAPE treatment was also applied on the RNAs after nuclei isolation and protein removal”.*

Minor comments:

3.	In panel a of Figure S3, nucleotide modification differences are hard to follow. This could be improved by including the percentage values within the bars.

*Response:*

*We thank the referee for this suggestion. We have added the percentage values in the panel a*

*of Figure S3 in our revised manuscript accordingly.*

4.	Lines 88 and 89: provide the full names of nucleotides for the abbreviations As, Cs etc.

*Response:*

*We thank the referee for this suggestion. We have added the full name of each nucleotide in*

*our revised manuscript, accordingly.*

5.	Typo in Line 198: "reactivits"

*Response:*

*We apologize for this typing error, which we have corrected in our revised manuscript.*

6.	Lines 569 and 891: "semi-qPCR" should be "semiquantitative RT-PCR".

*Response:*

*We thank the referee for this suggestion. We have changed "semi-qPCR" to "semiquantitative*

*RT-PCR" in our revised manuscript, accordingly.*

Reviewer #2:

Liu et.al use chemical probing (SHAPE) to probe the genome-wide nuclear and cytoplasmic structures

of Arabidopsis with and without proteinase treatment.

Their main claims are

1.	Nuc-SHAPE generates in vivo RNA structure landscape with high accuracy.

2.	Nuclear structures are globally different than cytosolic structures

3.	2-nt single stranded RNA is present upstream at splice sites (ss) and sufficient to regulate splicing

0.	The polyadenylation signal (PAS) and poly(A) sites are generally open and that promotes recognition.

These claims are of broad interest but I find that the data is weak, the necessary controls are not included, and the conclusions are not adequately supported. Here are main specific concerns for each of the above four claims.

*Response:*

*We thank the Referee for their appreciation that our work is: “.....of broad interest”. We are grateful for the referee’s constructive suggestions to help us strengthen our manuscript. We have fully addressed all their comments by undertaking extensive new experiments and new data analysis as follows.*

(1) There isn't convincing evidence that the RNA structure models are highly accurate or reproducible. The authors show good data for very abundant, extremely structured RNAs (U1, U12). There are no positive control mRNA structures and positive control structures in low abundant messages.

*Response:*

*U1 and U12 snRNA are two constitutively expressed RNA molecules in the nucleus. Their secondary structures are well documented in many previous studies [1, 2]. The structure of U1 and U12 snRNA are conserved among different species and are stably folded in living cells [1, 2]. Thus, we selected U1 and U12 snRNA as positive controls for our nuclear RNA secondary structure probing.*

*Following the Referee’s suggestions, we have also now included mRNA structures as additional positive controls, alongside U1 and U12 snRNAs. We have added the TPP (thiamin pyrophosphate) riboswitch on the pre-mRNA of Arabidopsis THIC gene as an additional positive control for our in vivo nuclear mRNA structure probing (Rebuttal Figure 3, new Figure S6). The TPP riboswitch is an evolutionarily conserved RNA structure among many different plant species [3]. Our in vivo SHAPE reactivities agree well with the evolutionary conserved structure of the TPP riboswitch (Rebuttal Figure 3, new Figure S6). Moreover, we also added the secondary structures of the 5’UTR regions for two Arabidopsis mRNAs, PSBA (ATCG00020) and GRP3S (AT2G05380) as additional positive controls for cytosolic mRNA structure probing (Rebuttal Figure 3, new Figure S6). These two in vivo secondary structure models of PSBA and GRP3S were determined by other in vivo RNA structure probing methods (DMS/SHAPE-RT and DMS/SHAPE-LMPCR) from a previous study [1]. Both DMS/SHAPE-RT and DMS/SHAPE-LMPCR were designed for individual RNA structure probing [1]. As shown in Rebuttal Figure 3, the SHAPE reactivity profiles from our genome-wide study agree very well with those previously published RNA structure models. All these positive controls provide further support to our conclusion that our new method can accurately capture the in vivo structure information of mRNAs.*

*Rebuttal Figure 3 (new Figure S6). Comparison of SHAPE reactivity profiles with previously published mRNA secondary structure models.*

*a, The evolutionary conserved RNA secondary structure model of Arabidopsis THIC pre-mRNA (TPP (thiamin pyrophosphate) riboswitch) before splicing [3]. In vivo SHAPE reactivity values from our Nuc-SHAPE-Structure-Seq agree well with the structure model. Nucleotides were colour-coded according to in vivo SHAPE reactivity values (SHAPE reactivity 0.6-1.0 marked in red, 0.3-0.6 marked in yellow, 0-0.3 marked in green). b, A in vivo RNA secondary structure model in the 5’UTR of Arabidopsis chloroplast gene PSBA (ATCG00020). SHAPE reactivity values from our SHAPE-Structure-Seq agree with the previously-identified structure model. The AU-box (denoted by circled nucleotides) was found to be single-stranded, which is consistent with previous study [1]. c, A in vivo secondary structure model in the 5′UTR of Arabidopsis GRP3S (AT2G05380) determined by DMS-LMPCR in a previous study [1]. SHAPE reactivity values from our SHAPE-Structure-Seq agree with the structure model.*

The authors need to provide justification for using the coverage cut off they did. Some essential comparisons and data quality metrics are missing:

-	The Pearson correlation of the SHAPE signal for replicates across each message (or a given window of nucleotides) as a function of the massage abundance; that would be one way to choose an appropriate threshold for the minimum coverage.

-	The Pearson correlation of the SHAPE signal per message (or window) plotted for in vivo vs untreated, biological replicates, and nuclear vs cytoplasmic RNAs.

*Response:*

*We thank the referee for these suggestions and have added the essential comparisons and data quality metrics, accordingly.*

*We have calculated the Pearson correlation coefficient (PCC) of the SHAPE signal between replicates for each mRNA and plotted the corresponding average PCC as a function of the RT-stop read coverage (RT-stop counts per nucleotide) (Rebuttal Figure 4, new Figure S5). The PCC values increase when the RT-stop read coverage increases (Rebuttal Figure 4, new Figure S5). The average PCC values are 0.71 and 0.75 for mRNAs with more than one RT-stop count per nucleotide in in vivo nuclear and cytosolic SHAPE libraries, respectively (Rebuttal Figure 4, new Figure S5), which indicates that SHAPE signals of mRNAs with the cut-off of over one RT-stop count per nucleotide, provide high correlation between different replicates.*

*Furthermore, we have also calculated the Pearson correlation coefficient (PCC) of the RT-stop counts of each transcript for SHAPE treated vs no-SHAPE treated libraries, biological replicates, and nuclear vs cytosolic mRNAs (Rebuttal Figure 5). High correlations (average PCC=0.71 for nuclear SHAPE libraries and 0.75 for cytosolic SHAPE libraries) were observed between the two replicates of SHAPE treated libraries, which again indicates the high reproducibility of SHAPE signals between the biological replicates. In contrast, very low correlations were observed between SHAPE treated and untreated libraries (average PCC=0.31 for nuclear SHAPE libraries and 0.38 for cytosolic SHAPE libraries), which indicates the specificity of SHAPE modification. The correlations of SHAPE signals between nuclear SHAPE libraries and cytosolic SHAPE libraries (average PCC = ~0.64) were significantly lower than SHAPE signals between the replicates (average PCC=0.71 and 0.75), which agrees with our observation of global structural differences between nuclear and cytosolic mRNAs (Rebuttal Figure 5).*

***Rebuttal Figure 4 (new Figure S5). Average Pearson correlation coefficient (PCC) of the***

***SHAPE signals between the two biological replicates for both in vivo nuclear and cytosolic SHAPE libraries for mRNAs with different RT-stop reads counts.***

*Average Pearson correlation coefficient (PCC) values are shown for transcripts with different sequencing depth (reverse transcription stop (RT-stop) counts per nucleotide) in both in vivo nuclear (****a****) and cytosolic SHAPE libraries (****b****).*

*Rebuttal Figure 5. Average Pearson correlation coefficient (PCC) of RT-stop counts profiles between different libraries.*

*The PCC (Pearson correlation coefficient) of RT-stop counts of mRNAs with more than one RT-stop count per nucleotide was calculated between in vivo SHAPE treated vs no-SHAPE treated libraries, between two replicates of in vivo SHAPE treated libraries and between nuclear SHAPE libraries and cytosolic SHAPE libraries. The PCC distributions of each comparison were shown.*

(2) This claim has very little support. The authors are averaging thousands of messages and find that one condition (nuclear vs cytoplasmic) gives them something on the order of 0.01-0.05 signal difference in average SHAPE reactivity (Fig2). That could easily be an artifact (e.g. RNA binding proteins that they didn't manage to get rid of during the proteinase treatment or simply a reproducibility artifact). For comparison, in Fig 1., the authors bin all reactivates that fall between 0.0 and 0.3 as a single color (green) to suggest that bases with signal between 0.0 and 0.3 are equivalent. However, in Fig2 the authors claim that 0.05 differences in signal is important and enough for a major result of their paper (i.e nuclear structure is different than cytoplasmic). The argument is not convincing. Further, it is very strange that the coding sequence close to the 5'UTR behaves differently than the coding sequence close to the 3'UTR, this doesn't make biological sense.

*Response:*

*We thank the referee for their comment.*

*The average SHAPE reactivity comparison in Figure 2a allowed us to directly compare the global difference between nuclear mRNAs and cytosolic mRNAs, which further led us to compare the SHAPE reactivity difference for different genic regions (Figure 2b,c). These comparisons provide us with a general view of the overall SHAPE reactivity differences between nuclear and cytosolic mRNAs, which is quite common for meta-gene analysis of global RNA structure features [4-6]. While the order of SHAPE reactivity difference has a small numeric value, our statistics test demonstrates a strongly significant difference in the values.*

*In Figure 1, we colour-coded different SHAPE reactivity values. Since RNA structure is dynamic, the range of SHAPE reactivity reflects the different degrees of single-strandedness of nucleotides. A high reactivity denotes the nucleotide which is likely to be more single-stranded, whereas a low reactivity denotes the opposite. We coded the SHAPE reactivity range (0-0.3) with the same colour as a means to visually present our data in a more accessible way that highlights the range of SHAPE reactivity for one particular RNA, and does not set a cut-off threshold for further analysis.*

*As shown in Figure 2, we observed higher SHAPE reactivities upstream of start codons, lower SHAPE reactivities downstream of start codons, and higher SHAPE reactivities at stop codons in our cytosolic SHAPE-Structure-Seq libraries (Figure 2c,d). These RNA structural features were also observed in previous studies [4, 7-9]. We acknowledge the referee has raised an interesting question: the biological interpretation of the SHAPE reactivity features observed near translation start and stop codons. High accessibility at the nucleotides immediately upstream of the start codon was reported to have a major role in regulating translation efficiency [10, 11]. A previous in vitro study suggested that mature mRNAs might require strong structures downstream of the start codon for increasing the 40S subunit “dwell time” [12]. Lower SHAPE reactivities downstream of start codons implied that stronger structures downstream of the start codon might relate to ribosome pausing in vivo. The single-stranded RNA structure feature at the stop codon was also observed in a previous RNA structure study and was suggested to facilitate translation termination [13]. Further studies could investigate these tentative biological functions in detail.*

-	Fig 4a shows the reactivity of 70nt region in AT5G56870. There appears to be only 1 or 2 bases reactive and these bases are hyper reactive. This doesn't look like a structure profile; it looks like a profile of two hyper reactive bases. Are the authors suggesting that all the rest 68nt are paired? Where is the structure model for the wt gene in a window of 100-200nt ? Are these structures supported by other metrics such as evolutionary conservation or thermodynamic stability (e.g. ScanFold) ?

*Response:*

*We thank the referee for their comments.*

*SHAPE reagent modifies single-stranded nucleotides. Therefore, if a nucleotide is base paired, the SHAPE reactivity value tends to be equal or close to “0”. Since RNA structure is dynamic, the range of SHAPE reactivity reflects the different degrees of single-strandedness of nucleotides. Therefore, smaller SHAPE reactivity values flanking the 5’ss represent relatively small probability of SHAPE modification. Following the Referee’s suggestion, we plotted the SHAPE reactivity profile from -50 to +50 (Rebuttal Figure 6). The upstream of the exon-intron junction are more single-stranded as compared to the region across the exon-intron junction (Rebuttal Figure 6). We have also followed the referee’s suggestion and predicted the thermodynamically stable RNA structure model (Minimum Free Energy (MFE) RNA structure) (Rebuttal Figure 7a). As shown, the -1 and -2 positions of 5’ss are single- stranded in the MFE RNA structure model (Rebuttal Figure 7a), which agrees with the higher SHAPE reactivity values.*

*Moreover, we have assessed the conservation of the RNA structure model across 5’ss. We have extracted the sequence information of different plant species (Arabidopsis thaliana, Arabidopsis halleri, Arabidopsis lyrata, Brassica oleracea, Brassica rapa and Raphanus sativus) from Ensembl Plants and NCBI nt/nr database. As shown in Rebuttal Figure 7b, the stem structures surrounding 5’ss shows very high sequence conservation among different species. Notably, two of the few sequence variations display GU-AU or AU-GU base-pairing alterations, which does not disrupt the stem structure (Red arrows in Rebuttal Figure 7b), further suggesting the conservation of the structure. These results suggest the stem structure surround 5’ss is likely to be evolutionarily conserved among these species.*

***Rebuttal Figure 6. SHAPE reactivity profiles across the 5’ss of the first intron of AT5G56870.***

*Rebuttal Figure 7. Thermodynamically stable RNA structure model across the 5’ss of AT5G56870 intron 1 and sequence conservation analysis across the 5’ss.*

*a, The minimum free energy (MFE) RNA structure model was generated for the exon-intron region of AT5G56780 intron 1 by using ViennaRNA Package [14]. The unpaired probability values are colour-coded and aligned to the MFE RNA structure model. Different colours on different nucleotides represent the probability values of being unpaired. 5’ss and the stems were denoted. b, Multiple sequence alignment is shown for the exon-intron region of AT5G56780 intron 1. The black boxes indicated the regions folded into stems in the MFE RNA structure model of Arabidopsis thaliana as shown in (a) panel. Red arrows denote the sequence variations showing GU-AU or AU-GU base-pairing alteration, which does not disrupt the stem structures.*

-	Fig 4b is interesting but a big conclusion is made based on too few experiments. For example, the authors didn't mutate any bases upstream of the GAG. This experiment requires a high throughput mutagenesis and testing multiple messages in order to conclude that the two nucleotides upstream of the ss are most important for regulating splicing, which seems unlikely to be generally true. The contribution of the surrounding sequence and structure must be rigorously tested by mutagenesis and splicing assay of multiple messages.

*Response:*

*We thank the referee for their suggestions. We have performed extensive new experiments by adding the same functional validations for two additional genes AT1G08450 and AT3G08930 (Rebuttal Figure 1 (New Figure S10)). Moreover, following the Referee’s suggestions, we have also designed mutations on more positions including nucleotides upstream of the GAG to assess their effects on splicing (Rebuttal Figure 1 (New Figure S10), Rebuttal Figure 2b,d (Revised Figure 4b,d)). For all these three genes, only the mutations which led to the single-strandedness at -1 and -2 positions of 5’ss can significantly restore the splicing events (Rebuttal Figure 1 (New Figure S10), Rebuttal Figure 2b,d (Revised Figure 4b,d)). Hence, our results confirmed that the two-nucleotide single-stranded RNA structure feature at -1 and -2 positions upstream of 5’ss is sufficient to regulate splicing.*

-	Fig.S7 - the authors clam there is no sequence difference of the splicing motifs but that is a subjective claim. The authors make a big deal out of differences in SHAPE signal of 0.05 but there is an equivalent amount of sequence variation, for example in S7a, the bottom (unspliced) events have an enrichment for AC that is not present in the top GU (spliced) sites.

*Response:*

*We thank the referee for their comment. In Figure S9 (original Figure S7), there is very minor sequence composition difference across splice sites between spliced and unspliced introns. For -1 and -2 positions, a conserved AG dinucleotide was observed for both spliced and unspliced introns. To further confirm our conclusion that the SHAPE reactivity difference at -1 and -2 position of 5’ss is not due to the sequence composition difference, we further compared the SHAPE reactivity between spliced and unspliced introns with the same 5’ss sequence (from -3 to +5 position) (Rebuttal Figure 8). Similarly, SHAPE reactivities at the -1 and -2 positions upstream of 5’ss were significantly higher in spliced events than those in unspliced events for each group with the same 5’ss sequence (Rebuttal Figure 8). Therefore, these results further confirmed our conclusions.*

*Rebuttal Figure 8. SHAPE reactivity profiles across 5’ splice site of spliced and unspliced events with the same 5’ splice site sequences.*

*The three most frequently used 5’ splice site sequences (AAGGTAAT, GAGGTAAA and AAGGTAAG, from -3 to +5 positions of 5’ splice site) were used. SHAPE reactivity profiles of spliced and unspliced introns with exactly the same 5’ splice site sequence were plotted and compared. The -1 and -2 positions at 5’ splice site are highlighted in yellow.*

(3) It is difficult to make conclusions out of these results. For example, the authors show in Fig. 5g that indeed 20/26% of the PAS site and PolyA sites are in stems. Are these sites less functional or are these sites equally good motifs as the ones that are in loops? Also fig.5a, why do the control sites have lower SHAPE reactivity baseline than any of the PolyA sites? The control sites control for sequence, they should have the same baseline, unless there is protein contamination or something else not structure (perhaps not enough sites were used in each bin to accurately reflect the average behavior), which is creating a higher SHAPE baseline for the polyA sites. The presence of multiple potential artifacts make it difficult, or impossible, to interpret the "bump" in SHAPE reactivity.

*Response:*

*We thank the referee for this comment. Figure 5g shows the results of in silico prediction of RNA structure across poly(A) sites. Most of the PAS (polyadenylation signal, 80%) sites and cleavage sites (74%) were folded into single-stranded RNA structures while only a small proportion (20% for PAS sites, 26% for cleavage sites) folded as double-stranded stem structures. Many factors affect the poly(A) site selection, such as phase separation and Pol II pausing [15, 16]. Here, we showed that RNA structure is also associated with the selection of poly(A) sites for a large proportion of genes.*

*Figure 5a shows that sequence at cleavage sites and -20nt upstream of cleavage sites (corresponding to PAS sites) have significant higher average SHAPE reactivities than the flanking sequences, however, this significant structural feature was not observed in control sites. The population size (4,636 for in vivo condition and 5,354 for deproteinized condition) is high enough for reflecting the average behaviour. Similar SHAPE reactivity patterns were observed in our deproteinized library, indicating this SHAPE reactivity pattern was not due to protein contamination (Figure 5b). Thus, this RNA structure feature comprising two close-by single-stranded regions is associated with polyadenylation. The observation that SHAPE reactivities at control sites are relatively lower than poly(A) sites suggests that the 3’ end of nascent mRNA before polyadenylation occurs tends to be more accessible than other genic regions in living cells [17]. The more single-stranded region in the 3’ end of nascent mRNA before processing are required to facilitate the recruitment of 3’ processing machinery later [17]. Additionally, we would like to emphasize that our study revealed a strong association between RNA structure and poly(A) site selection. While RNA structure is likely to be one of the major factors, we acknowledge it is not the only factor affecting poly(A) site selection.*

*References*

*1.	Kwok CK, Ding Y, Tang Y, Assmann SM, Bevilacqua PC. Determination of in vivo RNA structure in low-abundance transcripts. Nat Commun. 2013;4(2971).*

*2.	Pomeranz Krummel DA, Oubridge C, Leung AK, Li J, Nagai K. Crystal structure of human spliceosomal U1 snRNP at 5.5 A resolution. Nature. 2009;458(7237):475-480.*

*3.	Wachter A, Tunc-Ozdemir M, Grove BC, Green PJ, Shintani DK, Breaker RR. Riboswitch control of gene expression in plants by splicing and alternative 3' end processing of mRNAs. Plant Cell. 2007;19(11):3437-3450.*

*4.	Spitale RC, Flynn RA, Zhang QC, Crisalli P, Lee B, Jung JW, Kuchelmeister HY, Batista PJ, Torre EA, Kool ET, Chang HY. Structural imprints in vivo decode RNA regulatory mechanisms. Nature. 2015;519(7544):486-490.*

*5.	Incarnato D, Neri F, Anselmi F, Oliviero S. Genome-wide profiling of mouse RNA secondary structures reveals key features of the mammalian transcriptome. Genome Biol. 2014;15(10):491.*

*6.	Sun L, Fazal FM, Li P, Broughton JP, Lee B, Tang L, Huang W, Kool ET, Chang HY, Zhang QC. RNA structure maps across mammalian cellular compartments. Nat Struct Mol Biol. 2019.*

*7.	Ding Y, Tang Y, Kwok CK, Zhang Y, Bevilacqua PC, Assmann SM. In vivo genome-wide profiling of RNA secondary structure reveals novel regulatory features. Nature. 2014;505(7485):696-700.*

*8.	Wan Y, Qu K, Zhang QC, Flynn RA, Manor O, Ouyang Z, Zhang J, Spitale RC, Snyder MP, Segal E, Chang HY. Landscape and variation of RNA secondary structure across the human transcriptome. Nature. 2014;505(7485):706-709.*

*9.	Kertesz M, Wan Y, Mazor E, Rinn JL, Nutter RC, Chang HY, Segal E. Genome-wide measurement of RNA secondary structure in yeast. Nature. 2010;467(7311):103-107.*

*10.	Wan Y, Qu K, Ouyang Z, Kertesz M, Li J, Tibshirani R, Makino DL, Nutter RC, Segal E, Chang HY. Genome-wide measurement of RNA folding energies. Mol Cell. 2012;48(2):169-181.*

*11.	Cannone JJ, Subramanian S, Schnare MN, Collett JR, D'Souza LM, Du Y, Feng B, Lin N, Madabusi LV, Muller KM, et al. The comparative RNA web (CRW) site: an online database of comparative sequence and structure information for ribosomal, intron, and other RNAs. BMC Bioinformatics. 2002;3(2).*

*12.	Hinnebusch AG, Ivanov IP, Sonenberg N. Translational control by 5'-untranslated regions of eukaryotic mRNAs. Science. 2016;352(6292):1413-1416.*

*13.	Shabalina SA, Ogurtsov AY, Spiridonov NA. A periodic pattern of mRNA secondary structure created by the genetic code. Nucleic Acids Res. 2006;34(8):2428-2437.*

*14.	Lorenz R, Bernhart SH, Honer Zu Siederdissen C, Tafer H, Flamm C, Stadler PF, Hofacker IL. ViennaRNA Package 2.0. Algorithms Mol Biol. 2011;6(26).*

*15.	Fang X, Wang L, Ishikawa R, Li Y, Fiedler M, Liu F, Calder G, Rowan B, Weigel D, Li P, Dean C. Arabidopsis FLL2 promotes liquid-liquid phase separation of polyadenylation complexes. Nature. 2019;569(7755):265-269.*

*16.	Anamika K, Gyenis A, Poidevin L, Poch O, Tora L. RNA polymerase II pausing downstream of core histone genes is different from genes producing polyadenylated transcripts. PLoS One. 2012;7(6):e38769.*

*17.	Yang Q, Doublie S. Structural biology of poly(A) site definition. Wiley Interdiscip Rev RNA. 2011;2(5):732-747.*

**Second round of review**

**Reviewer 2**

Overall, the quality of the genome-wide data is low. In rebuttal Fig.5 the mean person correlation coefficient is ~ 0.7. This means that r2= 0.49, in other words the SHAPE signal variation in one biological sample explains only 49% of the signal variation in the other biological sample. The authors need to make sure that an RNA structure model constrained with these SHAPE data are reproducible. Importantly, the authors should compare 1. Genome-wide structure model at splice sites (or other sites of interest) generated with no SHAPE constrains to the same model generated with SHAPE constrains from biological replicate 1 and biological replicate 2. Would they get the same results as Main Fig3 using predicted structures with SHAPE constrains? It is key to demonstrate the results in Main Fig3 in a different way and to show that the SHAPE data is indeed essential for these results (i.e. that -1 and -2 nt positions are open for spliced events).

Figure 2A/B is not convincing and not appropriate for the point the authors are trying to make. By eye there is no difference in the average of the SHAPE reactivity in nuclear vs cytoplasmic(2A), and even if statistically significant, the difference is very small. This figure should be removed because nothing can be concluded from it. Even if there were a difference in the average SHAPE reactivity in all bases of all RNAs, that alone can have many interpretations (e.g. chemical reactivity differences in the nuclear and cytoplasmic, etc.). Indeed, structure changes don’t necessarily change the average signal, they change the specific distribution of the signal across nucleotides (as some bases become open in one structure but paired in another). Finally, as the distribution of signal is not well correlated between biological replicates (r2= 0.49), any small differences are meaningless.

The authors addressed some of my points but not all. The specific mutations they make are very clean and convincing, but it is in the context of an engineered stem loop not an endogenous one and in my view a single example is not enough to make genome-wide conclusions. Here are more specific points:

Fig. 5 a- why do the control lines look so flat and the base line is different than the test samples? The authors response was inadequate. In fig 2A/B the authors interpret a small difference in the average reactivity but in Fig.5a the authors cannot account for a much larger difference between the base line of their control and their test samples? It seems the authors “cherry pick” which differences to interpret without good reasoning. These results also have no follow up and are based on averaging over many mRNAs, all the evidence for the polyA is weak and that should be a supplementary point.

Rebuttal Fig 6 and Fig7a did not address my concerns. In Fig.6 , why are all the bases -50 to -44 , with SHAPE signal of exactly 1.0? This doesn’t make sense unless the authors have cut off the y-axes scale or performed some normalization that caps the signal at 1.0 but that should be clearly stated and justified. Also, the region shown in 6a should correspond exactly to the region shown in 7a (which starts earlier at around -80nt). It is important to show the raw data for the very reactive loop at ~60nt as well. Rebuttal

Fig.7a should be color coded by SHAPE reactivities, it doesn’t make sense to suddenly change the color scale and show purely computational prediction. It should be the same color scale as used in other figures (Main Fig 1-5, SHAPE scale) and the structure should be derived from the SHAPE data.

The conclusions derived from the specific mutations (Fig4) are interesting and exciting. However, the authors need to strengthen the data to support generalizing these results. For example, if they look at all the endogenously spliced events, exactly how many are predicted to have an open -1 and -2 nt positions? How many are outliers? Is there an explanation for the outliers? Inversely for the unspliced events. Fig3 currently provides weak evidence, showing that, on average, all spliced events tend to have higher SHAPE reactivity at the -1 and -2nt but how does this average break down to individual endogenous messages?

**Authors Response**

**Point-by-point responses to the reviewers’ comments:**

Comment 1:

Overall, the quality of the genome-wide data is low. In rebuttal Fig.5 the mean person correlation coefficient is ~ 0.7. This means that r2= 0.49, in other words the SHAPE signal variation in one biological sample explains only 49% of the signal variation in the other biological sample.

*Response:*

*We understand the reviewer’s concern. There is a bit confusion regarding the previous rebuttal Fig.5 which was to show the PCCs of reverse transcription stops (RT-stops) between +SHAPE libraries and -SHAPE libraries and between the biological replicates. The +SHAPE libraries contains both SHAPE modification information and noise background (e.g. random reverse transcription stalling). Therefore, to derive the SHAPE signal (SHAPE reactivity), the noise background was subtracted from the +SHAPE libraries by using -SHAPE libraries as control. The average PCC values for this SHAPE signal (SHAPE reactivity) are 0.87 and 0.89 for mRNAs with more than one RT-stop count per nucleotide in in vivo nuclear and cytosolic SHAPE libraries, respectively (Rebuttal Figure 1 (revised Figure S5)), which indicates the high reproducibility of the SHAPE signals between biological replicates. Since the SHAPE reactivity eliminates noise background such as random reverse transcription stalling, the PCCs of the SHAPE reactivity is higher than the PCCs of RT-stop counts between biological replicates. We have revised previous Figure S5 to clarify this point (Rebuttal Figure 1 (revised Figure S5)).*

*Rebuttal Figure 1 (revised Figure S5). Average Pearson correlation coefficient (PCC) of SHAPE reactivities between the two biological replicates of the in vivo nuclear and cytosolic SHAPE-Structure-Seq libraries for mRNAs with different RT-stop reads counts.*

*Average Pearson correlation coefficient (PCC) values are shown for transcripts with different sequencing depth (reverse transcription stop (RT-stop) counts per nucleotide) in both in vivo nuclear (a) and cytosolic SHAPE-Structure-Seq libraries (b). The dash lines indicate the threshold of more than one RT-stop count per nucleotide.*

The authors need to make sure that an RNA structure model constrained with these SHAPE data are reproducible. Importantly, the authors should compare 1. Genome-wide structure model at splice sites (or other sites of interest) generated with no SHAPE constrains to the same model generated with SHAPE constrains from biological replicate 1 and biological replicate 2. Would they get the same results as Main Fig3 using predicted structures with SHAPE constrains? It is key to demonstrate the results in Main Fig3 in a different way and to show that the SHAPE data is indeed essential for these results (i.e. that -1 and -2 nt positions are open for spliced events).

*Response:*

*We thank the reviewer for these suggestions for strengthening our results. To fully address the reviewer’s concerns, we performed all the analysis the reviewer suggested.*

*Firstly, we plotted the SHAPE reactivity profiles for each replicates of the in vivo SHAPE libraries. As shown in Rebuttal Figure 2a and 2b, for both spliced and unspliced events, the SHAPE reactivity profiles of the two biological replicates are highly consistent with each other (Rebuttal Figure 2a,b) as well as our global SHAPE reactivity profiles in main Figure 3a. This further confirms the high reproducibility of the in vivo SHAPE reactivity between the two biological replicates in our libraries.*

*Furthermore, we performed RNA structure folding with (SHAPE constrained) and without SHAPE reactivity (non-SHAPE constrained) across the 5’ splice site (5’ss) for both spliced and unspliced events. The unpaired probability, derived from RNA structure folding, represents the average level of single-strandedness among all possible conformations in folding ensemble. Thus, we summarized and plotted the average unpaired probability of each position across 5’ss (Rebuttal Figure 2c,d). Consistent with the SHAPE reactivity profiles, we found that, for SHAPE-constrained structures of the spliced events, the average unpaired probability at the -1 and -2 positions were much higher than their neighboring positions (Rebuttal Figure 2c), while this phenomenon is absent in the unspliced events (Rebuttal Figure 2d, Mann-Whitney test p values are 5.3e-05 and 2.2e-16 for -1 and -2 positions of replicate 1, 3.4e-05 and 2.2e-16 for -1 and -2 positions of replicate 2, in the comparison between spliced and unspliced events). These results further indicate a higher level of single-strandedness at the -1 and -2 positions of the spliced events compared to the unspliced ones. The SHAPE-constrained structures for both biological replicates (red and blue lines) display similar patterns (Rebuttal Figure 2c,d), which indicates the high agreement between biological replicates. Notably, the average unpaired probabilities across the 5’ss in non-SHAPE constrained RNA structure folding are very different from the SHAPE-constrained structures for the spliced events (Rebuttal Figure 2c,d). This result is consistent with our previous studies and others that the in vivo RNA structure (SHAPE constrained structure) tend to be more single-stranded than the in silico predicted RNA structure (non-SHAPE constrained structure) [1-3]. It also agrees with the observation that the RNA structure tends to be more flexible and single-stranded in living cells [4, 5].*

*Rebuttal Figure 2. SHAPE reactivity and unpaired probability profiles of the two biological replicates across 5’ splice site.*

*SHAPE reactivity profiles of the two biological replicates across 5’ss were plotted for spliced events (a) and unspliced events (b). SHAPE reactivity constrained and non-SHAPE constrained RNA structure folding was performed. Unpaired probability profiles across 5’ss derived from the SHAPE constrained RNA structures and the non-SHAPE constrained RNA structures were shown for spliced events (c) and unspliced events (d). The 100nt sequences flanking 5’ss were used in RNA structure folding by RNAfold in Vienna package [3]. The -1 and -2 positions upstream of 5’ss are shaded in grey.*

Comment 2:

Figure 2A/B is not convincing and not appropriate for the point the authors are trying to make. By eye there is no difference in the average of the SHAPE reactivity in nuclear vs cytoplasmic (2A), and even if statistically significant, the difference is very small. This figure should be removed because nothing can be concluded from it. Even if there were a difference in the average SHAPE reactivity in all bases of all RNAs, that alone can have many interpretations (e.g. chemical reactivity differences in the nuclear and cytoplasmic, etc.). Indeed, structure changes don’t necessarily change the average signal, they change the specific distribution of the signal across nucleotides (as some bases become open in one structure but paired in another). Finally, as the distribution of signal is not well correlated between biological replicates (r2= 0.49), any small differences are meaningless.

*Response:*

*We understand the reviewer’s concern. We have removed Figure 2a,b and revised the manuscript accordingly. Please see our highlighted parts in the revised manuscript on lines 160-178, 361-374 and 851-853.*

*Regarding the reproducibility of our SHAPE signals, please see our response to comment 1, where our SHAPE signals are highly reproducible.*

Comment 3:

The authors addressed some of my points but not all. The specific mutations they make are very clean and convincing, but it is in the context of an engineered stem loop not an endogenous one and in my view a single example is not enough to make genome-wide conclusions. Here are more specific points:

Fig. 5 a- why do the control lines look so flat and the base line is different than the test samples? The authors response was inadequate. In fig 2A/B the authors interpret a small difference in the average reactivity but in Fig.5a the authors cannot account for a much larger difference between the base line of their control and their test samples? It seems the authors “cherry pick” which differences to interpret without good reasoning. These results also have no follow up and are based on averaging over many mRNAs, all the evidence for the polyA is weak and that should be a supplementary point.

*Response:*

*We thank the reviewer for their appreciation that “The specific mutations they make are very clean and convincing”.*

*As the reviewer pointed out, we would emphasize that the analysis of polyadenylation associated RNA structure indeed serves as a supplementary point in our manuscript where we claimed that we found a genome-wide association between a SHAPE reactivity pattern and polyadenylation. We did not draw any further conclusions beyond this statement in our manuscript. This genome wide association is based on both the average SHAPE reactivity pattern (Figure 5a) and the SHAPE reactivity patterns of individual mRNAs (Figure 5c). We found two single-stranded regions (at about -20nt upstream of poly(A) sites and across poly(A) sites) based on the statistic evaluation (Fisher’s exact test, P-value = 3.1e-14 and 3.6e-6 for these two regions) (Figure 5a). No such SHAPE reactivity pattern was observed in control sites, i.e. control line is flatter than the true poly(A) site (Figure 5a).*

*To further strengthen our result, we plotted the SHAPE reactivity profiles for each biological replicate. Both SHAPE reactivity patterns and corresponding statistic evaluation agree well between the two replicates (Rebuttal Figure 3) as well as the patterns observed in Figure 5a.*

*To confirm whether our observed average feature represents the features of individual genes, we also showed the SHAPE reactivities across poly(A) site of individual mRNAs in heatmap (Figure 5c). As shown in Figure 5c, our heatmap presentation of SHAPE reactivities across poly(A) sites clearly demonstrated that the conventional PAS (PolyA Signal) sites and cleavage sites have higher SHAPE reactivities than the flanking regions for individual transcripts (Figure 5c). By performing SHAPE constrained RNA structure folding, we also found PAS sites and cleavage sites have lower base-pairing probability (high unpaired probability) for individual transcripts (Figure 5f), which was concordant with their higher SHAPE reactivities (Figure 5c).*

*In addition, following the reviewer’s suggestion, we have revised our manuscript by adding “We also observed that the overall SHAPE reactivities in control sites were lower than those in true poly(A) sites, which suggested that the 3’ end of nascent mRNA before polyadenylation occurs tends to be more accessible than other genic regions in living cells. This is consistent with previous observations that single-stranded RNA features are required for recruitment of 3’ processing machinery [6]” on lines 489-493.*

*Rebuttal Figure 3. in vivo SHAPE reactivity profiles of both replicates for ture poly(A) sites and control sites.*

*The x-axis represents the relative position to the cleavage sites. Average SHAPE reactivities i n two regions (gray shaded) were significantly (p values = 5.72e-12 and 4.59e-10 for regions upstream of poly(A) site and across poly(A) site in replicate 1; p values = 1.60e-05 and 1.19e -06 for regions upstream of poly(A) site and across poly(A) site in replicate 2, Fisher’s exact t est) higher compared to flanking regions for true poly(A) sites but not for control sites.*

Comment 4:

Rebuttal Fig 6 and Fig7a did not address my concerns. In Fig.6 , why are all the bases -50 to -44 , with SHAPE signal of exactly 1.0? This doesn’t make sense unless the authors have cut off the y-axes scale or performed some normalization that caps the signal at 1.0 but that should be clearly stated and justified.

Also, the region shown in 6a should correspond exactly to the region shown in 7a (which starts earlier at around -80nt). It is important to show the raw data for the very reactive loop at ~60nt as well.

Rebuttal Fig.7a should be color coded by SHAPE reactivities, it doesn’t make sense to suddenly change the color scale and show purely computational prediction. It should be the same color scale as used in other figures (Main Fig 1-5, SHAPE scale) and the structure should be derived from the SHAPE data.

*Response:*

*Thanks for the referee’s comment. Our SHAPE reactivity values were rescaled to 0-1 after removing the outliers by 90% winsorization, which is the normalization methods used in previous genome-wide RNA structure studies [4, 5, 7]. This method is widely used in global RNA structure studies, which allows the comparisons among RNAs of different length [4, 5, 7, 8]. We described this in the Method part on lines 600-601. In addition, we have showed that our SHAPE reactivity profiles agree well with the U1 snRNA, U12 snRNA and previously reported mRNA structure models, which confirmed that our SHAPE reactivity can accurately reflect the in vivo RNA secondary structure (Figure 1, Figure S6).*

*The unpaired probability is the counterpart of SHAPE reactivity in RNA structure folding and reflects the thermodynamic status of folding ensemble. To address the reviewer’s previous concern regarding if the -1 and -2 positions are single-stranded in the thermodynamically stable structure (MFE RNA structure), we colour-coded the RNA structure model with unpaired probability in previous Rebuttal Fig.7a. Here, we added the colour-coded RNA model with SHAPE reactivity values according to the reviewer’s suggestion, which is shown in Rebuttal Figure 4. The SHAPE reactivities from -100 to 100 positions of the MFE RNA structure model were also shown in Rebuttal Figure 5.*

*Rebuttal Figure 4. Minimum free energy RNA structure at the 5’ss of AT5G56780 intron 1 folded by RNAfold.*

*The minimum free energy (MFE) RNA structure for the exon-intron region of AT5G56780 intron 1 was generated by RNAfold program in ViennaRNA Package. The in vivo SHAPE reactivity values are color-coded and aligned to the MFE RNA structure (SHAPE reactivity 0.6-1.0 marked in red, 0.3-0.6 marked in yellow, 0-0.3 marked in green). The -1 and -2 positions are all single-stranded in the MFE RNA structure.*

*Rebuttal Figure 5. The SHAPE reactivity values across the 5’ss of the first intron of*

*AT5G56870*

*The x-axis represents the relative positions across the 5’ss.*

Comment 5:

The conclusions derived from the specific mutations (Fig4) are interesting and exciting. However, the authors need to strengthen the data to support generalizing these results. For example, if they look at all the endogenously spliced events, exactly how many are predicted to have an open -1 and -2 nt positions? How many are outliers? Is there an explanation for the outliers? Inversely for the unspliced events. Fig3 currently provides weak evidence, showing that, on average, all spliced events tend to have higher SHAPE reactivity at the -1 and -2nt but how does this average break down to individual endogenous messages?

*Response:*

*We thank the reviewer for their appreciation that “The conclusions derived from the specific mutations (Fig4) are interesting and exciting” and for their valuable suggestions. We have performed further analysis to strengthen our conclusions.*

*We performed in vivo SHAPE-constrained RNA structure folding and classified the folded MFE (minimum free energy) RNA structure models according to the folding status of - 1 and -2 nt positions. For spliced events, about 71% were predicted to be single-stranded at both -1 and -2 positions and only 8% of the predicted MFE structures were double-stranded at these two positions (Rebuttal Figure 6, left panel). In contrast, only 11% of the unspliced events were predicted to be single-stranded at both -1 and -2 nt positions (Rebuttal Figure 6, right panel). Thus, the single-strandedness at the -1 and -2 positions upstream of 5’ss represents a general RNA structure feature of spliced events.*

*Rebuttal Figure 6. Summary of the folding status of -1 and -2 positions upstream of 5’ss for spliced and unspliced events.*

*The minimum free energy (MFE) RNA structures were generated by in vivo SHAPE constrained folding with RNAfold program in ViennaRNA Package [3]. The folding status of - 1 and -2 nt positions of all the MFE structures were summarized for both spliced and unspliced events.*

*We did observe a small proportion of outliers. About 8% of the spliced events were predicted to be double-stranded at -1 and -2 positions in MFE structure models (Rebuttal Figure 6, left panel). The -1 and -2 positions of about 11% of the unspliced events were predicted to be single-stranded in MFE structure models (Rebuttal Figure 6, right panel). Many previous individual studies indicated that splicing can be regulated by many different factors, such as splicing factors [9-11], mRNA modifications [12, 13] and Pol II elongation rate [14]. For example, AT2G16940 and AT4G13850, two unspliced outliers in our study, were found as the targets of splicing suppressor AtPTB1 and AtPTB1 [9]. The splicing of AT4G02725 was suggested to be promoted by two splicing factors SC35 and SCL in Arabidopsis [10]. It is not surprise that there were some outliers since RNA structure is not the only factor affecting splicing. The relationship between other factors and RNA structure features will be an interesting question which requires new hypotheses and further study in the future.*

*In addition, to address the reviewer’s concern on whether the average SHAPE reactivity feature can represent the SHAPE reactivity features of individual mRNAs, we further plotted the SHAPE reactivities for each individual mRNAs with heatmaps (Rebuttal Figure 7 (new Figure S10)). As shown in Rebuttal Figure 7, significant patterns of higher SHAPE reactivities at -1 and -2 positions were observed among most spliced events but not unspliced ones, which is consistent with the average SHAPE reactivity pattern observed in Figure 3a. Taken together, our new results strongly support that the single-strandedness at -1 and -2 positions upstream of 5’ss of spliced events is a general phenomenon at whole-genome scale.*

*Rebuttal Figure 7 (new Figure S10). Heatmaps showing the SHAPE reactivities across 5’ss for spliced and unspliced events.*

*In vivo SHAPE reactivities across 5’ss were color coded for each individual mRNAs and shown in heatmap. The gradient color from light to dark red represents SHAPE reactivities from low to high.*

*References:*

*1.	Ding Y, Tang Y, Kwok CK, Zhang Y, Bevilacqua PC, Assmann SM. In vivo genome-wide profiling of RNA secondary structure reveals novel regulatory features. Nature. 2014;505(7485):696-700.*

*2.	Deng H, Cheema J, Zhang H, Woolfenden H, Norris M, Liu Z, Liu Q, Yang X, Yang M, Deng X, et al. Rice In Vivo RNA Structurome Reveals RNA Secondary Structure Conservation and Divergence in Plants. Mol Plant. 2018;11(4):607-622.*

*3.	Lorenz R, Bernhart SH, Honer Zu Siederdissen C, Tafer H, Flamm C, Stadler PF, Hofacker IL. ViennaRNA Package 2.0. Algorithms Mol Biol. 2011;6(26.*

*4.	Spitale RC, Flynn RA, Zhang QC, Crisalli P, Lee B, Jung JW, Kuchelmeister HY, Batista PJ, Torre EA, Kool ET, Chang HY. Structural imprints in vivo decode RNA regulatory mechanisms. Nature. 2015;519(7544):486-490.*

*5.	Rouskin S, Zubradt M, Washietl S, Kellis M, Weissman JS. Genome-wide probing of RNA structure reveals active unfolding of mRNA structures in vivo. Nature. 2014;505(7485):701-705.*

*6.	Yang Q, Doublie S. Structural biology of poly(A) site definition. Wiley Interdiscip Rev RNA. 2011;2(5):732-747.*

*7.	Sun L, Fazal FM, Li P, Broughton JP, Lee B, Tang L, Huang W, Kool ET, Chang HY, Zhang QC. RNA structure maps across mammalian cellular compartments. Nat Struct Mol Biol. 2019.*

*8.	Zubradt M, Gupta P, Persad S, Lambowitz AM, Weissman JS, Rouskin S. DMS-MaPseq for genome-wide or targeted RNA structure probing in vivo. Nat Methods. 2017;14(1):75-82.*

*9.	Simpson CG, Lewandowska D, Liney M, Davidson D, Chapman S, Fuller J, McNicol J, Shaw P, Brown JW. Arabidopsis PTB1 and PTB2 proteins negatively regulate splicing of a mini-exon splicing reporter and affect alternative splicing of endogenous genes differentially. New Phytol. 2014;203(2):424-436.*

*10.	Yan Q, Xia X, Sun Z, Fang Y. Depletion of Arabidopsis SC35 and SC35-like serine/arginine-rich proteins affects the transcription and splicing of a subset of genes. PLoS Genet. 2017;13(3):e1006663.*

*11.	Morton M, AlTamimi N, Butt H, Reddy ASN, Mahfouz M. Serine/Arginine-rich protein family of splicing regulators: New approaches to study splice isoform functions. Plant Sci. 2019;283(127-134.*

*12.	Roundtree IA, He C. Nuclear m(6)A Reader YTHDC1 Regulates mRNA Splicing. Trends Genet. 2016;32(6):320-321.*

*13.	Hu J, Manduzio S, Kang H. Epitranscriptomic RNA Methylation in Plant Development and Abiotic Stress Responses. Front Plant Sci. 2019;10(500.*

*14.	Godoy Herz MA, Kornblihtt AR. Alternative Splicing and Transcription Elongation in Plants. Front Plant Sci. 2019;10(309.*

**Third round of review**

**Referee 2**

1. It is important not to mislead the reader. Please use the correlation coefficient that is most appropriate, and that is the SHAPE signal- i.e. what you are actually using to constrain your models, i.e. r= 0.7. Please show it as r2 and don’t use the word “great” correlation, that should be reserved if explaining >90% variation. It would be best to state the correlation without adding subjective interpretation of how good it is, let the readers decide for themselves.

2. Rebuttal figure 2, 4, 5, and7 look good and add a lot to the manuscript.

3. It is very perplexing why in Rebuttal Fig.3, the overall Shape reactivity at ALL nucleotides is lower for control sites. Please add an explanation for this. State how many sites you are using for test and control. Can you exclude the effect of protein binding here? What is the result in the de-protonated samples?

4. Please make sure to directly address the possibility that this striking result for the (-1 and -2 ) positions is not due changes in SHAPE reactivity because of a protein factor (perhaps a splicing factor) binding in cells at these positions, which causes them to be over-reactive. Do you see the exact same result (Rebuttal Fig 7) when you deprotonate ?

This is an interesting study, I think the most critical point to strengthen the authors conclusions is to separate the effect of SHAPE reactivity on protein binding verses RNA structure.

**Authors Response**

**Point-by-point responses to the reviewers’ comments:**

In response to the Referee 2’s concerns, we thank the Referee for their suggestions. Overall, we generated deproteinzed libraries in parallel in our work to eliminate the effects from protein binding. The detailed responses to the Referee 2’s comments are as follows:

Comment 1:

1. It is important not to mislead the reader. Please use the correlation coefficient that is most appropriate, and that is the SHAPE signal- i.e. what you are actually using to constrain your models, i.e. r= 0.7. Please show it as r2 and don’t use the word “great” correlation, that should be reserved if explaining >90% variation. It would be best to state the correlation without adding subjective interpretation of how good it is, let the readers decide for themselves.

*Response:*

*We have added r2 instead of r in our new supplementary Figure S5. The r2 of the correlation coefficient are 0.76 and 0.79 for mRNAs with more than one RT-stop count per nucleotide in in vivo nuclear and cytosolic SHAPE libraries, respectively. We have never used the word “great” correlation in our manuscript. Instead, we used the word “high” correlation according to the general statistics where the r2 value over 0.7 is generally considered strong effect size (Moore, D. S., Notz, W. I, & Flinger, M. A. (2013). The basic practice of statistics (6th ed.). New York, NY: W. H. Freeman and Company. Page (138)).*

Comment 2:

2. Rebuttal figure 2, 4, 5, and 7 look good and add a lot to the manuscript.

*Response:*

*We thank the referee for their comment. We have added Rebuttal Figure 2, 4, 5 and 7 into our revised manuscript as new supplementary Figures S11, S13, S12 and S10, respectively.*

Comment 3:

3. It is very perplexing why in Rebuttal Fig.3, the overall Shape reactivity at ALL nucleotides is lower for control sites. Please add an explanation for this. State how many sites you are using for test and control. Can you exclude the effect of protein binding here? What is the result in the de-protonated samples?

*Response:*

*In our previous rebuttal letter, we have added an explanation for this structure feature and included it in the manuscript “We also observed that the overall SHAPE reactivities in control sites were lower than those in true poly(A) sites, which suggested that the 3’ end of nascent mRNA before polyadenylation occurs tends to be more accessible than other genic regions in living cells. This is consistent with previous observations that single-stranded RNA features are required for recruitment of 3’ processing machinery”. We have also added the number of sites in the figure legend for Figure 5 in our revised manuscript as “The numbers of control sites are 4,575 and 983 for in vivo and deproteinized conditions, respectively. The numbers of poly(A) sites are 3,077 and 551 for in vivo and deproteinized conditions, respectively.”.*

*The corresponding result in the deproteinized libraries was shown in Figure 5b and 5e, which is very similar to the observation in the in vivo libraries (Figure 5a and 5d). This indicates that this RNA structure feature was not due to the effect of protein binding.*

Comment 4:

4. Please make sure to directly address the possibility that this striking result for the (-1 and -2) positions is not due changes in SHAPE reactivity because of a protein factor (perhaps a splicing factor) binding in cells at these positions, which causes them to be over-reactive. Do you see the exact same result (Rebuttal Fig 7) when you deprotonate?

*Response:*

*Significantly high SHAPE reactivities were also observed at -1 and -2 positions of 5’ss in our deproteinized libraries (Figure 3b), which indicated that this RNA structural feature is not affected by protein factor binding. In addition, SHAPE modifies the single-stranded nucleotides. Therefore, the nucleotides interacted with a protein factor cannot be over-reactive.*
